# A disinhibitory mechanism biases *Drosophila* innate light preference

**DOI:** 10.1038/s41467-018-07929-w

**Published:** 2019-01-10

**Authors:** Weiqiao Zhao, Peipei Zhou, Caixia Gong, Zhenhuan Ouyang, Jie Wang, Nenggan Zheng, Zhefeng Gong

**Affiliations:** 1 Department of Neurology of the Second Affiliated Hospital, Zhejiang University School of Medicine, Hangzhou, Zhejiang 310058 China; 2 Department of Neurobiology, Key Laboratory of Medical Neurobiology of the Ministry of Health of China, Key Laboratory of Neurobiology, Zhejiang University School of Medicine, Hangzhou, Zhejiang 310058 China; 3 Qiushi Academy for Advanced Studies, Zhejiang University, Hangzhou, Zhejiang 310007 China

## Abstract

Innate preference toward environmental conditions is crucial for animal survival. Although much is known about the neural processing of sensory information, how the aversive or attractive sensory stimulus is transformed through central brain neurons into avoidance or approaching behavior is largely unclear. Here we show that *Drosophila* larval light preference behavior is regulated by a disinhibitory mechanism. In the disinhibitory circuit, a pair of GABAergic neurons exerts tonic inhibition on one pair of contralateral projecting neurons that control larval reorientation behavior. When a larva enters the light area, the reorientation-controlling neurons are disinhibited to allow reorientation to occur as the upstream inhibitory neurons are repressed by light. When the larva exits the light area, the inhibition on the downstream neurons is restored to repress further reorientation and thus prevents the larva from re-entering the light area. We suggest that disinhibition may serve as a common neural mechanism for animal innate preference behavior.

## Abstract (web summary)

The reorientation response of *Drosophila* larva to light is an innate behaviour. Here the authors identify a pair of GABAergic neurons that mediate a disinhibitory mechanism that regulates the larval reorientation response.

## Introduction

When choosing between two alternative conditions, animals such as *Drosophila* larva reorientates when facing unfavorable conditions but maintain unchanged directions when facing favorable conditions^1–3^. The choice behavior involves transforming sensory input into motor action of reorientation. In vertebrates, although the brain regions or neurons that are responsible for sensory information processing and motor control have been relatively well mapped^4,5^, the cellular and molecular mechanism underlying sensorimotor transformation in central brain has only been reported in a few cases, such as cutaneous or olfactory input-induced locomotion in xenopus and lamprey^6^.

In *Drosophila* larva, Bolwig’s organs, i.e. the photoreceptors, regulate larval avoidance response to light in both laboratory and outdoor experiments^7–12^. Bolwig’s organs directly send projections into the larval optical neuropil (LON) in central brain and synapse on visual local neurons and visual projection neurons^13^. These downstream neurons, including visual local neurons such as lOLP (local optic lobe pioneer) neurons, and visual projection neurons such as *pdf* neurons and 5^th^ lateral neuron in clock circuit as well as PVL09 neurons (posterior-ventro-lateral neuron 09), have been reported to be involved in various forms of larval light navigational behaviors^7,9,11,12,14^. At the level of motor control, neurons in *Drosophila* larval SEZ (subesophageal zone) have been suggested to command larval reorientation behavior in light avoidance^15^. But the neuronal circuitry that bridges the gap between upstream visual processing neurons and downstream turning command neurons has been left blank^16^. How the visual signal is transformed into an avoidance behavior remains largely elusive.

Disinhibition is a central mechanism that serves in various neural functions, such as sensory signal processing^17–19^, selection of motor programs^20,21^, memory expression^22^, and the switch between wake and sleep status^23^. In a disinhibitory microcircuit, the inhibition on downstream inhibitory neurons is supposed to be tonic, whereas the inhibition on upstream inhibitory neurons should be phasic^20,24,25^. This enables the efficient temporal control of the excitability of the downstream neurons.

Here we show that *Drosophila* larval avoidance to light is gated in a disinhibitory manner. We propose that disinhibition is the underlying mechanism for the initiation of choice action and subsequent securement of the correct choice in animal choice behavior.

## Results

### Larval light avoidance requires inhibition of LRIN^R13B07^s

**Fig. 1:**
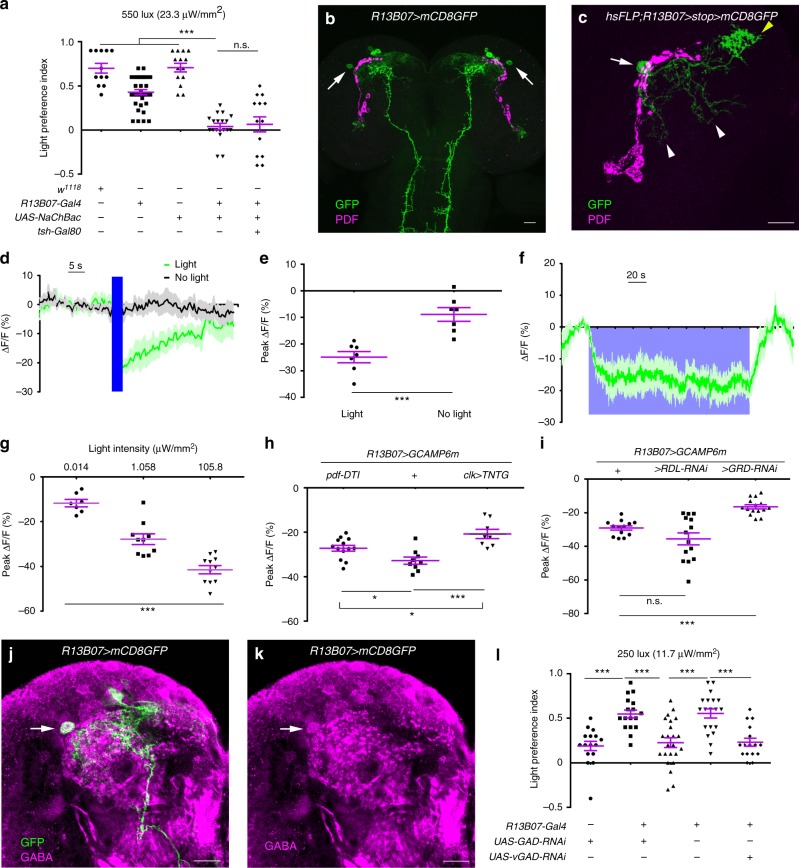
Light inhibition of GABAergic LRINR13B07s in brain is required for larval light avoidance. **a** Activation of *R13B07-Gal4* neurons in brain abolishes larval avoidance to white light at 550 lux (23.3 μW/mm^2^). **b** Expression pattern of *R13B07-Gal4* in larval brain. Arrows point to the LRIN^R13B07^s. **c** Morphology of single LRIN^R13B07^. Arrow, yellow and white arrow heads, respectively, point to the cell body, axonal termini and dendrites of LRIN^R13B07^. **d**–**e** LRIN^R13B07^s are inhibited by 470 nm light at intensity of 1.058 μW/mm^2^. **e** is the statistics of the peak calcium responses in **d**. **f** Sustained inhibition on LRIN^R13B07^s by light. 470 nm light at intensity of 0.001 μW/mm^2^ was used. *n* = 3. **g** Extent of inhibition on LRIN^R13B07^s is increased as light intensity increases. 470 nm light at intensity of 0.014 μW/mm^2^, 1.058 μW/mm^2^, and 105.8 μW/mm^2^ was used for 3 s. **h** Blocking *clk-LexA* labeled neurons or knocking out *pdf* neurons reduces the inhibition of LRIN^R13B07^s by light. 470 nm light at intensity of 105.8 μW/mm^2^ was used for 3 s. The genotypes of *pdf-DTI/UAS-GCAMP6m;R13B07-Gal4/+*, *UAS-GCAMP6m/+*;*R13B07-Gal4/+* and *clk-LexA/UAS-GCAMP6m;R13B07-Gal4/ LexAop-TNTG* are used. **i** Knocking down GRD expression in LRIN^R13B07^s reduced the inhibition of LRIN^R13B07^s by light. 470 nm light at intensity of 1.058 μW/mm^2^ was used for 3 s. The genotypes are *UAS-GCAMP6m*/*+*;*R13B07-Gal4*/*+*, *UAS-GCAMP6m/UAS-RDL-RNAi;R13B07-Gal4/+* and *UAS-GCAMP6m/UAS-GRD-RNAi;R13B07-Gal4/+*, respectively. **j**–**k** Single slice view of anti-GABA staining against LRIN^R13B07^. Arrows point to the co-localization in cell body of LRIN^R13B07^. **j** is the channel for GABA in **i**. **l** Knocking down expression of GAD or vGAT in LRIN^R13B07^s enhances larval avoidance to white light at 250 lux (11.7 μW/mm^2^). The blue bars in **d** and violet block in **f** indicate periods of light stimulation. Scale bars, 20 μm in **b**–**c** and **j**–**k**. n.s. not significant, *P < 0.05, **P < 0.01, ***P < 0.001, *t*-test in **e**, one-way ANOVA in **g**, one-way ANOVA with Tukey’s post hoc test in **a**, **h**, **i**, and **l**. Error bars, SEMs. Source data of **a**, **d**–**i**, **l** are provided as a source data file

To discover neurons that inhibit *Drosophila* larval reorientation in light avoidance, we crossed Gal4 lines with *UAS-NaChBac* which increases neuronal excitability and tested the larvae in a light/dark choice assay at a light intensity of 550 lux (23.3 μW/mm^2^). One Gal4 line of *R13B07-Gal4* that labeled about seven to ten interneurons in the anterior part of each larval brain hemisphere and a group of neurons in posterior part of VNC (ventral nerve cord), in addition to the Rh6-positive photoreceptors in peripheral nervous system, demonstrated an abolished larval light avoidance (Fig. 1a, b, Supplementary Fig. 1, Supplementary Fig. 2a–c). This defect was not rescued by introduction of *tsh-Gal80* that specifically represses Gal4 activity in VNC, suggesting that larval light avoidance did not involve the *R13B07-Gal4* labeled neurons in the VNC (Fig. 1a, Supplementary Fig. 1a–c). As hyperactivating Rh6-positive neurons alone did not affect larval light avoidance, they could also be excluded (Supplementary Fig. 2d). So it should be the neurons in brain hemispheres that repressed larval light avoidance.

As activation of these neurons negatively regulated light avoidance, we hypothesized that these neurons might be inhibited by light. We then tested the responses of the brain hemisphere neurons to light in calcium imaging. Among these neurons, only one pair of anterio-laterally localized neurons that each possessed a comprehensive dendritic region posterior-medial to the cell body and a dense sytGFP labeled axonal arborization region anterio-medial to the dendrites (Fig. 1c, Supplementary Fig. 1e, f), was strongly inhibited by blue light stimulation (Fig. 1d, e). As these neurons turned out to be inhibitory later, we named these *R13B07-Gal4* labeled light repressed inhibitory neurons LRIN^R13B07^s. The inhibition persisted as light was on for up to 3 min (Fig. 1f). When light intensity increased, this inhibition was strengthened (Fig. 1g). As the dendritic regions of the neurons were adjacent to the axonal projection area of lateral clock neurons outlined by anti-PDF, we reasoned that they might receive visual inputs from the clock neurons (Fig. 1c)^12,14,26^. We made use of a *R43D05-LexA* line that was generated using a fragment of the promoter of *clock(clk)* gene. This line labeled *pdf* neurons and neurons that were morphologically similar to the fifth lateral neurons (5^th^ LN), DN1 (dorsal neuron 1), DN2 (dorsal neuron 2), in addition to three clusters of putatively immature neurons in each brain hemisphere (Supplementary Fig. 3). When the *clk-LexA* labeled neurons were blocked with TNTG, a presynaptic inhibitor of neurotransmission, the extent of the inhibition was significantly reduced, suggesting that the inhibition at least partially originated from the *clk-LexA* labeled neurons (Fig. 1h). Specifically ablating *pdf* neurons using *pdf-DTI* (DTI, diphtheria toxin) could also relieve the light-induced inhibition, but obviously not as effective as inhibiting *clk-LexA* neurons (Fig. 1h). We next asked which receptor was used by LRIN^R13B07^s to receive the inhibitory input. Knocking down in LRIN^R13B07^s the expression of a GABA/Glycine receptor GRD^27,28^, but not of another GABA receptor RDL^29^, could efficiently reduce the extent of inhibition (Fig. 1i). This meant that the inhibition of LRIN^R13B07^s by light was mediated by the GRD receptor.

### LRIN^R13B07^s are GABAergic

As the LRIN^R13B07^s were inhibited by light and the activation of LRIN^R13B07^s suppressed larval light avoidance, we reasoned that these light inhibited neurons might inhibit other neurons that promote light avoidance. We co-stained the antibody against GABA, the most widely used inhibitory neurotransmitter, with *R13B07-Gal4* to test whether they were indeed inhibitory. The anti-GABA signal indeed co-localized well with the cell bodies of the LRIN^R13B07^s (Fig. 1j, k). LRIN^R13B07^s signal did not co-localize with anti-ChAT (choline acetyltransferase) that marks the cholinergic neurons, but about three other *R13B07-Gal4* labeled neurons in each larval brain hemisphere did (Supplementary Fig. 1g–i). This was verified by our observation that introduction of *Cha-Gal80* reduced the number of *R13B07-Gal4* labeled neurons by about three in each brain hemisphere (Supplementary Fig. 1j). The remaining labeling of axonal termini of photoreceptor neurons might be due to insufficient repression on Gal4 activity by *Cha-Gal80*. On the other hand, *vGlut-Gal80*, which was assumed to be expressed in glutamatergic neurons, did not obviously affect the expression of *R13B07-Gal4* in larval brain (Supplementary Fig. 1k). We then tested the role of GABA in LRIN^R13B07^s in larval light avoidance using the light/dark choice assay. When we knocked down expression of GABA synthesizing enzyme GAD (glutamate decarboxylase) or vesicular GABA transporter vGAT (vesicular GABA transporter) in the LRIN^R13B07^s, larval light avoidance was enhanced (Fig. 1l). It should be noted that we used relatively weaker light of 250 lux (11.7 μW/mm^2^) instead of 550 lux (23.3 μW/mm^2^) in the assay to reduce the level of light avoidance in control lines, thus making more space for further improvement in light preference index. Although the possibility that LRIN^R13B07^s were cholinergic or glutamatergic could not be completely excluded based on absence of labeling, they were the only *R13B07-Gal4* labeled neurons that were GABAergic and inhibited by light. Therefore, they should be the neurons that were responsible for larval light avoidance among all the *R13B07-Gal4* labeled neurons. As LRIN^R13B07^s were inhibitory neurons that were inhibited by light, larval light avoidance thus might be regulated by a disinhibitory mechanism^18–23^.

### LRIN^R13B07^s inhibit CLPN^R82B09^s

**Fig. 2:**
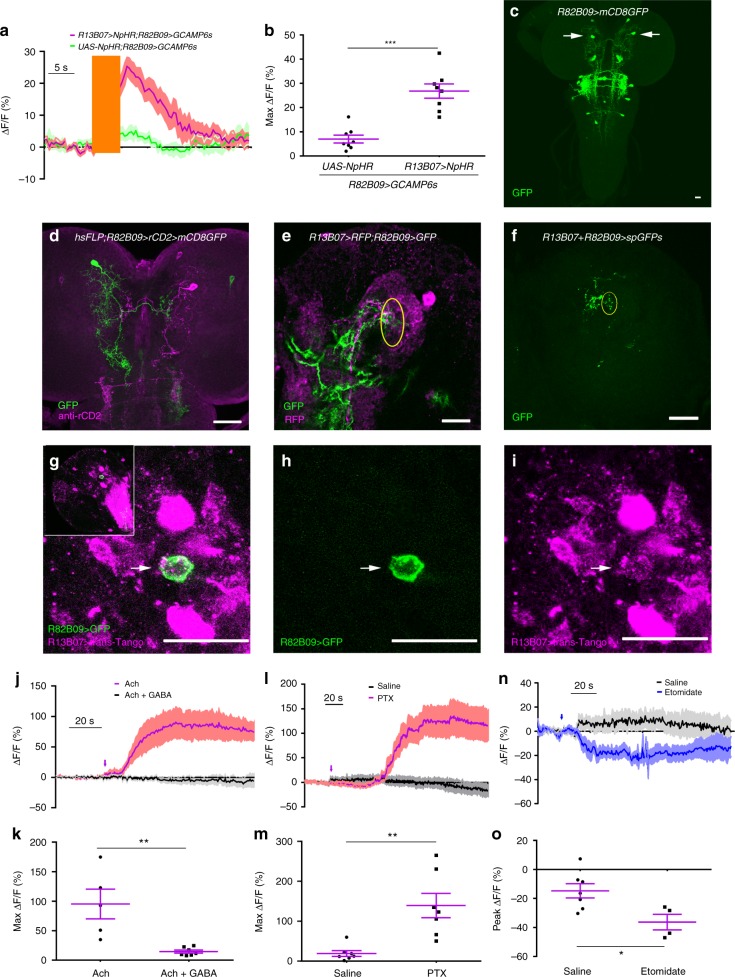
RDL on CLPN^R82B09^s mediates the inhibition from LRIN^R13B07^s **a**–**b**. Optogenetic inhibition of LRIN^R13B07^s allows excitation of CLPN^R82B09^s. **b** is the statistics of peak calcium responses in **a**. The orange bar indicates the 540 nm light stimulation. The genotypes are *R82B09-LexA/UAS-NpHR; LexAop-GCAMP6s/+* and *R82B09-LexA/UAS-NpHR; R13B07-Gal4/ LexAop-GCAMP6s*. **c** Expression pattern of *R82B09-Gal4* in larval brain. Arrows point to cell bodies of CLPN ^R82B09^s. **d** Morphology of single CLPN^R82B09^ labeled by *R82B09-Gal4*. **e** Co-localization between the dendrites of CLPN^R82B09^ and LRIN^R13B07^ in larval brain. Yellow circle outlines the overlapping region between LRIN^R13B07^ and CLPN^R82B09^. **f** GRASP signal between CLPN^R82B09^ and LRIN^R13B07^. Yellow circle outlines verified contact region. **g**–**i** trans-Tango probes CLPN^R82B09^ as immediate downstream of *R13B07-Gal4* labeled neuron. **g** is the merged version of **h** and **i**. trans-Tango signal is in magenta. GFP signal driven by *R82B09-Gal4* is in green. Arrows indicated the overlapping between trans-Tango signal and GFP signal that labels the cell body of a CLPN^R82B09^. Inset in **g** shows the position of the co-localization in larval brain hemisphere. *UAS-myrGFP,QUAS-mtdTomato(3xHA)/+;trans-Tango/+;R13B07-Gal4/R82B09-Gal4* is the genotype of the larva used. trans-Tango signals are reported by 3xHA. **j**–**k** Application of 100 mM GABA repressed the activation of CLPN^R82B09^s by 100 μM acetycholine. **h** is the statistics of peak calcium responses in **g**. **l**–**m** Application of 100 μM RDL antagonist picrotoxin allows excitation of CLPN^R82B09^s. **j** is the statistics of peak calcium responses in **i**. **n**–**o** Calcium response in CLPN^R82B09^s before and after etomidate application. Arrow indicates application of drug. **o** is the statistics of peak responses in **n**. Scale bars, 20 μm in **c**–**i**. Samples in **j**–**o** were prepared from larvae of genotype *R82B09-LexA;LexAop-GCAMP6s*. Arrows indicate application of drugs in **j**, **l**, and **n**. *P < 0.05, **P < 0.01, ***P < 0.001, *t*-test in **b**, **k**, **m**, and **o**. Error bars, SEMs. Source data of **a**–**b** and **j**–**o** are provided as a source data file

We next searched for the light avoidance promoting neurons downstream of LRIN^R13B07^s. Inhibition of the LRIN^R13B07^s using an optogenetic tool NpHR induced a robust calcium signal increase in one pair of central brain neurons labeled by *R82B09-Gal4* (Fig. 2a–c), suggesting that these neurons had been subjected to tonic inhibition from the LRIN^R13B07^s. These contralateral projecting neurons were then named as CLPN^R82B09^s. They were also labeled by *R82B09-LexA* and *R82B10-Gal4* (Supplementary Fig. 4a-c). The CLPN^R82B09^ had a small axonal arborization region in the medial contralateral brain hemisphere, a widespread dendritic region near the dendrites of the LRIN^R13B07^s, and a smaller dendritic arborization area in larval SEZ (Fig. 2d, Supplementary Fig. 4d–f). The dendrites of LRIN^R13B07^s were found to be overlapping with the dendrites of the CLPN^R82B09^s (Fig. 2e). We then used GRASP technique to confirm the contact between LRIN^R13B07^s and CLPN^R82B09^s. A strong GRASP signal was seen in the overlapping region (Fig. 2f, Supplementary Fig. 5). The putative synaptic contact was validated by trans-Tango^30^, a newly developed technique that can be used to probe downstream synaptic partners of neurons. By driving expression of trans-Tango with *R13B07-Gal4* to search for immediately downstream neurons, a large amount of cells were successfully marked (Supplementary Fig. 6a–b). Some of these cells were in the same region of CLPN^R82B09^s (Supplementary Fig. 6b). We then added *R82B09-Gal4* in the system to see if the trans-Tango signals driven by *R13B07-Gal4* overlap with the GFP signals that marked CLPN^R82B09^. As expected, co-localization was readily found (Fig. 2g–i). This result was in support of a direct dendrodendritic interaction between LRIN^R13B07^s and CLPN^R82B09^s, as driving trans-Tango using *R82B09-Gal4* alone did not yield any CLPN^R82B09^ signal (Supplementary Fig. 6c, d). As LRIN^R13B07^s were GABAergic, we reasoned that CLPN^R82B09^s should be subjected to direct GABAergic inhibition.

To confirm this hypothesis, we applied drugs to dissected and digested larval brain samples, in which CLPN^R82B09^s were disassociated and more susceptible to drug application. GABA could efficiently repress the excitation of CLPN^R82B09^s induced by acetycholine in calcium imaging (Fig. 2j, k). This result meant that CLPN^R82B09^s indeed subjected to GABAergic inhibition, in addition to cholinergic excitation. Furthermore, the application of RDL antagonist picrotoxin could strongly activate CLPN^R82B09^s (Fig. 2l, m), while RDL agonist etomidate could efficiently inhibit CLPN^R82B09^s (Fig. 2n, o). Together, these data suggested that CLPN^R82B09^s used RDL to receive GABAergic inhibitory input from LRIN^R13B07^s.

### Light responsive CLPN^R82B09^s control larval reorientation

**Fig. 3:**
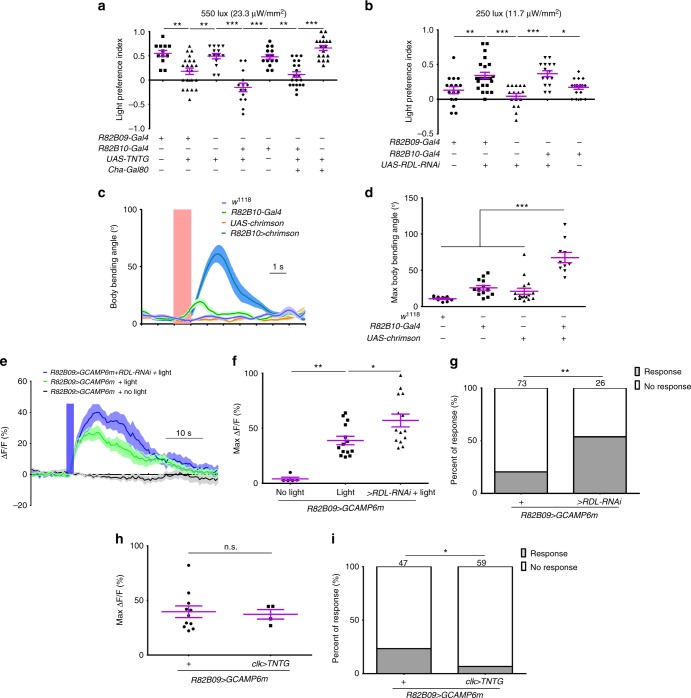
The CLPN^R82B09^s control head cast response to light. **a** Inhibiting CLPN^R82B09^s abolishes larval avoidance to white light at 550 lux (23.3 μW/mm^2^). **b** Knocking down RDL expression in CLPN^R82B09^s enhances larval avoidance to white light at 250 lux (11.7 μW/mm^2^). **c**–**d** Optogenetic stimulation of CLPN^R82B09^s evokes larval head cast. **d** is the statistics of head cast sizes in **c**. The pink bar indicates the period of optogenetic stimulation. **e**–**g** Knocking down RDL expression in CLPN^R82B09^s increases both amplitude and probability of CLPN^R82B09^s’ response to light in calcium imaging. The blue bar in **e** indicates 470 nm light stimulation at intensity of 10.58 μW/mm^2^. **f** is the statistics of peak responses in **e**. **h**–**i** Blocking *clk-LexA* neurons with TNTG does not affect the amplitude (**h**), but undermines the probability of CLPN^R82B09^s’ response to light in calcium imaging (**i**). One second 470 nm light stimulation at intensity of 10.58 μW/mm^2^ was used. In **e**–**i,** *R82B09>GCAMP6m* is the short for *UAS-GCAMP6m/+;R82B09-Gal4/+*. *R82B09>GCAMP6m+RDL-RNAi* is the short for *UAS-GCAMP6m/UAS-RDL-RNAi;R82B09-Gal4/+*. *clk>TNTG;R82B09>GCAMP6m* is the short for *clk-LexA/UAS-GCAMP6m; R82B09-Gal4/LexAop-TNTG*. Numbers above columns in **g** and **i** indicate sample sizes. n.s. not significant, *P < 0.05, **P < 0.01, ***P < 0.001, one-way ANOVA with Tukey’s post hoc test in **a**–**b**, **d**, and **f**, *t*-test in **h**, fisher’s exact test in **g** and **i**. Error bars, SEMs. Source data of **a**–**i** are provided as a source data file

We next examined if CLPN^R82B09^s were required for larval light avoidance. Blocking CLPN^R82B09^s by expression TNTG with either *R82B09-Gal4* or *R82B10-Gal4* abolished larval preference for darkness in the light/dark choice assay at 550 lux (23.3 μW/mm^2^) (Fig. 3a). Introduction of *Cha-Gal80* which removed Gal4 activity in all neurons except CLPN^R82B09^s did not rescue the defect (Fig. 3a and Supplementary Fig. 4c). Additionally, knocking down RDL expression in CLPN^R82B09^s enhanced the larval preference for darkness over light when a relatively weaker light intensity of 250 lux (11.7 μW/mm^2^) was used (Fig. 3b). On the other hand, when we expressed *UAS-Chrimson* with *R82B10-Gal4* to activate the CLPN^R82B09^s, robust larval head casts were observed (Fig. 3c, d). This could even be realized by optogenetic activation of a single CLPN ^R82B09^ (Supplementary Video 1). These results suggested that the CLPN^R82B09^s might control light-induced head cast. Indeed, our calcium imaging results showed that the CLPN^R82B09^s did respond to light stimulation (Fig. 3e, f). It is noted that the calcium transient could last for up to 40 s, whereas larval light avoidance response generally takes only at most a few seconds. The long duration of calcium response was likely due to dissection of larval body before calcium imaging that greatly changed the physiological environment of the imaged neurons. Knocking down RDL expression in the CLPN^R82B09^s not only increased the probability (see Methods for more details) (Fig. 3g), but also significantly improved the amplitude of the response (Fig. 3e, f). As a neuronal calcium transient usually reflects the accumulative effect of a bout of action potentials, the increased probability in calcium response reflected greater chance for burst of action potentials^31–33^. We further asked if the light input to CLPN^R82B09^s was also mediated by the known visual pathway neurons such as the *clk-LexA* labeled neurons and *pdf* neurons. Inhibiting *clk-LexA* neurons using TNTG significantly reduced the probability but not the amplitude of CLPN^R82B09^s’ light response in calcium imaging (Fig. 3h, i). However, ablating *pdf* neurons using *pdf-DTI* affected neither the probability nor the amplitude (Supplementary Fig. 7). This meant that at least part of the light signal transmitted to CLPN^R82B09^s was through the *clk-LexA* neurons and this part of signal might decide whether CLPN^R82B09^s responded to light or not, but not how strong the responses were. The above results together suggested that CLPN^R82B09^s controlled the light-induced head cast in light avoidance.

### Disinhibition of CLPN^R82B09^s facilitates larval head cast

**Fig. 4:**
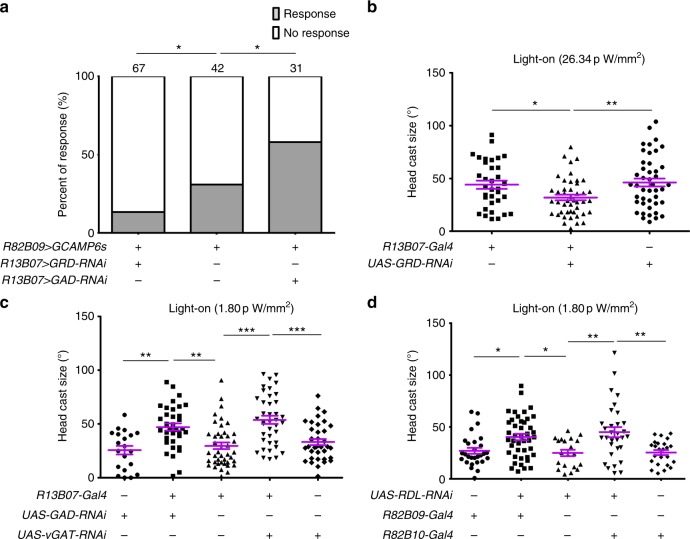
Larval reorientation at light-on is facilitated by disinhibition of CLPN^R82B09^s. **a** Knocking down GRD and GAD expression in LRIN^R13B07^s, respectively, reduces and increases the probability for CLPN^R82B09^s to respond to light in calcium imaging. One second 470 nm light stimulation at intensity of 1.058 μW/mm^2^ was used. Numbers above columns indicate sample sizes. Genotypes of larvae used are *R82B09-LexA/UAS-GRD-RNAi;LexAop-GCAMP6s/R13B07-Gal4*, *R82B09-LexA/+;LexAop-GCAMP6s/+* and *R82B09-LexA/UAS-GAD-RNAi;LexAop-GCAMP6s/R13B07-Gal4*, respectively. **b**–**d** Size of larval head cast at light-on in the light spot assay is reduced by downregulation of GRD in LRIN^R13B07^s (**b**), but is enhanced by down regulation of GAD/vGAT in LRIN^R13B07^s (**c)** or RDL in CLPN^R82B09^s (**d**). Light intensity in spot measured at 470 nm was 26.34 pW/mm^2^ for **b**, 1.80 pW/mm^2^ for **c**–**d**. *P < 0.05, **P < 0.01, ***P < 0.001, *χ*^2^-test with subgroup comparisons controlled by Benjamini-Hochberg method in **a**, one-way ANOVA with Tukey’s post hoc test in **b**–**d**. Error bars, SEMs. Source data of **a**–**d** are provided as a source data file

Despite that light inhibits LRIN^R13B07^s and LRIN^R13B07^s inhibit CLPN^R82B09^s, it was still not clear if the light-induced larval reorientation was regulated by disinhibition of CLPN^R82B09^s through inhibition of LRIN^R13B07^s. To confirm this hypothesis, we first tested whether the CLPN^R82B09^s’ response to light was regulated by the LRIN^R13B07^s- CLPN^R82B09^s inhibition. Knocking down GRD expression in LRIN^R13B07^s to reduce the light-induced inhibition on LRIN^R13B07^s could significantly reduce the probability for CLPN^R82B09^s to respond to light, while the amplitude of the response seemed to be unaffected (Fig. 4a and Supplementary Fig. 8). On the other hand, when we knocked down GAD expression in LRIN^R13B07^s to relieve the GABAergic inhibition on CLPN^R82B09^s, the probability for CLPN^R82B09^s to respond to light was significantly improved, although the amplitude of the response was also unaffected (Fig. 4a and Supplementary Fig. 8). These results were in consistence with the effects of blocking *clk-LexA* neurons as shown in Fig. 3f, g. It was probably because blockage of *clk-LexA* neurons reduced light inhibition on LRIN^R13B07^s that CLPN^R82B09^s were no longer efficiently disinhibited. In the case of ablating *pdf* neurons, light inhibition on LRIN^R13B07^s was also reduced but not as much as blocking *clk-LexA* neurons, so that the inhibition of CLPN^R82B09^s by LRIN^R13B07^s could still be relieved. Taken together, these results suggested that CLPN^R82B09^’s response to light was indeed gated by inhibition of LRIN^R13B07^s.

We next tested whether light-induced larval head cast was also regulated by the disinhibitory mechanism. We measured size of larval head cast in response to light-on using a light spot assay under a dim white light as larval head cast size saturated when light became perceivably high. We used relatively higher (26.34 pW/mm^2^, measured at 470 nm, see Methods) or lower (1.80 pW/mm^2^, measured at 470 nm) light intensity to elevate or lower the level of head cast size in controls, so as to make enough room for further decrease or increase in experimental groups. We first knocked down expression of GRD in the LRIN^R13B07^s to mitigate the inhibition of LRIN^R13B07^s by light. As shown in Fig. 4b, size of larval head cast upon light-on was significantly reduced in the GRD knockdown group as compared to that of the controls, at light intensity of 26.34 pW/mm^2^. This suggests that inhibition of the inhibitory LRIN^R13B07^s was indeed necessary for larval aversive response to light. On the other hand at relatively weaker light intensity of 1.80 pW/mm^2^, when GAD or vGAT was downregulated in LRIN^R13B07^s to reduce the GABAergic inhibitory input to CLPN^R82B09^s, size of light-on-induced larval head casts was significantly enhanced (Fig. 4c). In addition, knocking down RDL expression in CLPN^R82B09^s to relieve the inhibition on CLPN^R82B09^s also significantly enhanced size of larval head cast upon light-on at light intensity of 1.80 pW/mm^2^ (Fig. 4d). These results were consistent with our previous conclusion that disinhibtion of CLPN^R82B09^s through inhibiting LRIN^R13B07^s gated CLPN^R82B09^s’ response to light since CLPN^R82B09^s’ firing probability is positively related to the ratio of light-induced head cast over spontaneous head cast. As sizes of the light-induced head casts are usually larger than that of the spontaneous ones, CLPN^R82B09^s’ firing probability is positively related to the measured head cast size.

Next, to exclude the possibility that the increased head cast sizes resulted from the manipulations of neuronal activities even in absence of light, we performed the light spot assay with light constantly kept off, i.e. the light spot was actually dark. The differences in larval head cast in response to “light-on” were no longer observed (Supplementary Fig. 9a, b). Thus, the increased head cast sizes were indeed the outcomes of the interaction between the neuronal activities and light stimulation. Taken together, larval head cast response to light-on was indeed facilitated by disinhibition of CLPN^R82B09^s via LRIN^R13B07^s.

### Re-inhibition on CLPN^R82B09^s represses larval head cast

**Fig. 5:**
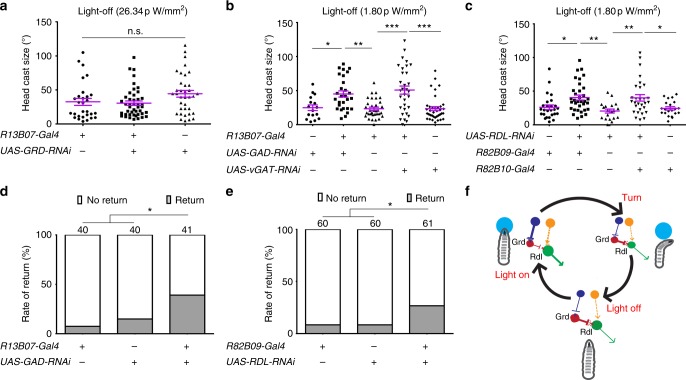
Larval reorientation at light-off is repressed by reinhibition of CLPN^R82B09^s. **a**–**c** Size of larval head cast at light-off in the light spot assay is unaffected by downregulation of GRD in LRIN^R13B07^s (**a**), but is enhanced by down regulation of GAD/vGAT in LRIN^R13B07^s (**b)** or RDL in CLPN^R82B09^s (**c)**. **d**–**e** Knocking down GAD expression in LRIN^R13B07^s (**d**) or RDL in CLPN^R82B09^s (**e**) increases the rate of larva returning to light after light exit. Number above the columns indicates the sample sizes. **f** A cartoon showing the hypothesized working mechanism of the disinhibitory pathway in larval light avoidance. The large light blue circles indicate light spots. The circles with arrows are red for LRIN^R13B07^, green for CLPN^R82B09^, dark blue for *clk-LexA* labeled neurons and orange for unspecified excitatory neurons upstream of CLPN^R82B09^s, respectively. Arrow heads indicate excitatory input. Bar heads indicate inhibitory input. The thickness of arrows indicates the strength of the inhibitory or excitatory effects. Light intensity in spot measured at 470 nm was 26.34 pW/mm^2^ for **a,** 1.80 pW/mm^2^ for **b**–**e**. n.s. not significant, *P < 0.05, **P < 0.01, ***P < 0.001, one-way ANOVA with Tukey’s post hoc test in **a**–**c**, *χ*^2^-test with subgroup comparisons controlled by Benjamini-Hochberg method in **d** and **e**. Error bars, SEMs. Source data of **a**–**e** are provided as a source data file

Because light inhibition on LRIN^R13B07^s was removed after the light was turned off (Fig. 1f), we speculated that the inhibition of LRIN^R13B07^s on CLPN^R82B09^s would be naturally restored once the larva had exited the light spot. Further head cast would therefore be inhibited. We then examined the role of LRIN^R13B07^s-CLPN^R82B09^s inhibition in head cast for larvae that had just exited the light spot. As expected, at relatively high light intensity of 26.34 pW/mm^2^, knocking down the light receiving receptor GRD in LRIN^R13B07^s did not affect larval head cast size upon light exit as the absence of GRD did not affect LRIN^R13B07^s-CLPN^R82B09^s inhibition in darkness (Fig. 5a). However, knocking down GAD or vGAT expression in the LRIN^R13B07^s could significantly improve size of head cast upon light exit (Fig. 5b), as could also be seen in the larvae with RDL downregulated in the CLPN^R82B09^s (Fig. 5c), at light intensity of 1.80 pW/mm^2^. The enhancement in larval head cast upon “light exit” was not seen if light was constantly kept off during the assay (Supplementary Fig. 9c, d). These results meant that the stimulatory effect of light on larval head cast could persist even after light went off. The immediate restoration of the inhibition on CLPN^R82B09^s prevented further head cast upon light exit.

As head cast could potentially bring the larva back into the light spot after it had left, the immediate restoration of the LRIN^R13B07^s-CLPN^R82B09^s inhibition might help to secure the “correct” choice of light escape by repressing the potential larval head cast. We then tested this assumption by examining the chance for the larva to return to light spot after the initial light escape. Knocking down GAD expression in LRIN^R13B07^s to prevent restoration of LRIN^R13B07^s-CLPN^R82B09^s inhibition indeed significantly increased the rate of larvae returning to light spot from no more than 15.00 to 39.02% (Fig. 5d, Supplementary Fig. 10). Similarly, knocking down RDL in the CLPN^R82B09^s also increased the rate from 8.33 to 26.67% (Fig. 5e). Thus, the restoration of inhibition on CLPN^R82B09^s could help improve larval light avoidance by repressing potential improper head cast after the initial light escape.

## Discussion

In this work, we discovered a disinhibitory neural mechanism that gated *Drosophila* larval head cast in presence and absence of light (Fig. 5f). LRIN^R13B07^s that exert a tonic inhibition on the larval head cast controlling CLPN^R82B09^s are inhibited by light to facilitate the head cast response. Once the larva escapes from light successfully, the inhibition on CLPN^R82B09^s is naturally restored to prevent further improper head casts, thus securing the success of light avoidance.

Such a disinhibitory mechanism has several roles. First, the inhibition on CLPN^R82B09^s represses larval behavioral response to light. This justifies the reduced larval responsiveness to very dim light that is usually safe. It also helps larvae to terminate behavioral response to light when necessary. Second, the inhibition of LRIN^R13B07^s by light ensures the specificity of larval orientation in response to light, as the inhibition on CLPN^R82B09^s prevents their excitation by non-visual stimulus. This specificity can be further enhanced if there exists another pathway for light to stimulate CLPN^R82B09^s^34^. Third, the opposite regulation of reorientation at transitions between light and darkness enhances larval preference for darkness over light. In addition to the explicit light avoidance facilitated by the head cast upon light stimulation, light avoidance is also enhanced by the inhibition of further potential head casts after light escape, in an implicit manner.

Compared with the recently reconstructed larval visual system that includes the first to third order neurons^13^, LRIN^R13B07^s and CLPN^R82B09^s should be at least the 3^rd^ or higher order neurons. For the known reconstructed second order visual neurons that likely project to region of LRIN^R13B07^s’ dendrites, all are cholinergic except for *pdf* neurons^13^. As *pdf* neurons carry only part of the inhibitory signal to LRIN^R13B07^s, those cholinergic neurons must activate some downstream inhibitory neurons that inhibit LRIN^R13B07^s. *pdf* neurons themselves may also inhibit LRIN^R13B07^s through other downstream neurons. Besides the second order neurons, one pair of third order glutamatergic visual projection local neurons also project to the region of LRIN^R13B07^s’ dendrites. They may channel part of the inhibition on LRIN^R13B07^s. As for CLPN^R82B09^s, they are disinhibited by light through the disinhibitory pathway. As they are also susceptible to cholinergic excitatory input (Fig. 2j, k), it is possible that CLPN^R82B09^s can also be activated by light through cholinergic neurons. On the output side, as parts of dendritic termini of CLPN^R82B09^s end in SEZ (Fig. 2d), a region that has been suggested to be crucial for motor control^15,16,35^, CLPN^R82B09^s may thus target onto the turning command neurons in SEZ to regulate larval head cast. It is likely that CLPN^R82B09^s also control larval head cast response to aversive sensory stimulus in other modalities, such as gustation or olfaction.

The observation that *pdf* neurons channel part of the light inhibition on LRIN^R13B07^s prompts us to reconsider the role of *pdf* neurons in larval light avoidance. It has been well established that light could entrain larval clock and induce photophobic behaviors through Bolwig’s organs. Downstream to Bolwig’s organs, *pdf* neurons were known to mediate the entraining of clock, but their roles in light avoidance has been on debate^7,12^. Based on our observation, *pdf* neurons do have the potential to affect larval light avoidance through mediating the light inhibition on LRIN^R13B07^s, although ablating *pdf* neurons did not significantly change CLPN ^R82B09^’s response to light. It is possible that their role in light avoidance cannot be readily detected, unless light signal goes through those non-pdf neurons is lessened.

One important property of LRIN^R13B07^s in the disinhibitory neural circuit is that it is sensitive to dim light, although the light inhibition does not saturate even at high light intensities. But at behavioral level, the size of the disinhibition regulated larval head cast in response to dark-to-light transition seemed to saturate at moderate light intensity. Therefore, when larvae choose a dark or dim condition over a brighter condition, the light avoidance must involve the LRIN^R13B07^s-CLPN^R82B09^s disinhibitory circuit. For example, for feeding larvae that digs deep into food, the contrast between the light outside and the dark inside may provide part of the driving force as the opaque food surrounding larval eyes makes an almost completely dark local environment. On the other hand, when larvae choose between bright and brighter light, larval head cast in both conditions may saturate so that light avoidance may not happen. If it does, the disinhibition of CPLNs should not be involved.

The disinhibitory control of light elicited larval turning behavior resembles the disinhibitory mechanism in vertebrates for motor program selection. In vertebrate, striatal neurons from motor cortex send inhibitory signal to inhibit pallidum neurons that exert tonic inhibition on spinal motor command neurons^20^. One advantage of this mechanism is the strict and precise control of motor initiation and termination. The adoption of disinhibitory mechanism in *Drosophila* larval motor control suggests that the higher level control of motor initiation is conserved among invertebrate and vertebrate.

As most innate preferences are realized though reorientation, we suggest disinhibition to be a common neural mechanism underlying animal innate preference behavior.

## Methods

### Fly culture and strains

All flies were raised at 25 °C on standard medium and 12 h:12 h light/dark cycles of culture^36^. The following fly strains were used in this work: *w*^*1118*^*, Rh5-eGFP(BL8600); Rh6-eGFP(BL7461), Rh6-Gal4(BL7464), GMR-myrRFP(BL7121), R13B07-Gal4(BL48545), R82B10-Gal4(BL46717), R82B09-Gal4(BL40133), R82B09-LexA(BL61613), clk-LexA(R43D05-LexA,BL54147), pdf-LexA*^37^*, Cha-Gal80, vGlut-Gal80, tsh-Gal80*^38^*, GMR-Gal80, UAS-TNTG*^39^*, UAS-NaChBac*^40^*, UAS-GCAMP6m(BL42748)*^41^*, UAS-mCD8-GFP(BL5137), UAS-myrGFP(BL7118), UAS-Chrimson(BL55135)*^42^*, UAS-NpHR(BL41753)*^43^*, UAS-sytGFP,UAS-Denmark(BL33064)*^44^*, UAS-mCD8-RFP(BL32218), UAS-myrGFP,QUAS-mtdTomato(3xHA)(X); trans-Tango (II)*^30^*, hsFLP, UAS-FRT-rCD2-FRT-mCD8GFP, UAS-FRT-stop-FRT-Chrimson, UAS-FRT-stop-FRT-sytGFP, UAS-GAD-RNAi(TH02214.N), UAS-vGAT-RNAi(THU4304), UAS-RDL-RNAi(TH02821.N), UAS-GRD-RNAi(THU3884), LexAop-myrGFP(BL32209), LexAop-TNTG, LexAop-GCAMP6s(BL44273)*^41^*, LexAop-CD4::GFP*_*11*_*;UAS-CD4::GFP*_*1-10*_^37,45^, *pdf-DTI*^37^. The RNAi lines were from Qinghua *Drosophila* Stock center.

### Light/dark choice assay for larval light preference

The protocol for test of larval light/dark preference was as previously reported^37^. In brief, 20 cleaned 3^rd^ instar larvae were placed on a 1.5% agar plate with one half covered with a taped lid. White light generated from a fluorescent light was placed above the plate to illuminate the uncovered half. Light intensity of 550 lux corresponded to 23.3 μW/mm^2^, 250 lux corresponded to 11.7 μW/mm^2^ at maximal readings (S401C, Thorlabs, Inc). After 2 min of adaptation on agar plate, larvae were aligned along the light/dark boundary and allowed to move freely for 10 min. Then the numbers of larvae in each half of agar plate were scored. Experimental temperature was at 25.5 °C.

Larval light preference index (PI) was calculated as:

PI = (number of larvae in the dark half–number of larvae in the light half)/(number of larvae in the dark half + number of larvae in the light half). PIs were shown as mean ± SEM.

### Light spot assay

The procedure of light spot assay was largely same as previously described^46^. Individual larva was first acclimated on an agar plate for 2 min in a dark room. The plate was rotated to re-orientate larva heading toward a light spot of 2 cm-in-diameter generated by a white light LED before start of test. The white light had one intensity peak at 450 nm with half width of 10 nm and another peak at 583 nm with half width of 65 nm. The light intensity of 1.80 pW/mm^2^ or 26.34 pW/mm^2^ measured at 470 nm (S120C, Thorlabs, Inc.) in light spot was used. The whole process from larval entering to exiting of the light spot was video recorded with an infrared high-resolution web camera from above. The lens was covered by an 850 nm infrared narrow-pass filter to prevent the disturbance of visible light on video. Three 850 nm LED lights that were evenly placed around the plate to illuminate the arena. Experimental temperature was kept as 25.5 °C. Videos were analyzed with the software SOS and custom scripts (see below for larval head cast analysis). For larval re-entry to light spot, a larva that had its head touched the light spot for at least once within 20 s after the initial exit was defines as a larva of returning.

### Optogenetics

Eggs of proper genotypes were laid on food supplied with 0.2 mM trans-retinal. For testing behavioral consequence of optogenetic activation of CLPN^R82B09^s with Chrimson, cleaned single 3^rd^ instar larva was allowed to crawl on a 1.5% agar plate. A 620 nm red-light pulse was delivered onto the larva in periods of straight forward locomotion. The process of larval locomotion was video recorded and processed with SOS software and custom scripts (see below for larval head cast analysis). For imaging CLPN^R82B09^’s response to optogenetic inhibition of LRIN^R13B07^s using NpHR, 540 nm green light was used (also see below for calcium imaging).

### Pharmacology

Larval brain samples were prepared following previously reported protocols but with modifications^47^. Individual 3^rd^ instar larva was removed from food and washed alternatively with ddH_2_O and 70% ethanol for 1 min. It was dissected in standard saline solution (128 mM NaCl, 2 mM KCl, 4 mM MgCl_2_⊡6H_2_O, 1.8 mM CaCl_2_, 36 mM sucrose, 5 mM HEPES, pH = 7.1) and the brain were transferred to 1.5 ml microcentrifuge tube with 500 μl standard saline containing 0.4 mg/ml protease (Sigma-Aldrich, P5147) and 0.1 mg/ml collagenase (Sigma-Aldrich, C0130). The brain was digested for ~3 h before being centrifuged at 1 rcf for 2.5 min. The digest solution was pipetted off and 100 μl Schnerder’s *Drosophila* Medium (Gibco, 21720–024) containing 10% FBS (Sera Pro, S601S-500) was added. Single brain was transferred into round shaped recording chamber (16 mm in diameter) filled with 1.5 ml standard saline for calcium imaging. To prevent the sample from moving during circulating, brain tissue was covered by a custom made stainless steel grids (mesh size 200, hole size 100 μm). During calcium imaging, drugs were added into the recording chamber using a circulating pump (LongerPump, BT100–2J). Drugs used included picrotoxin (Hellobio, HB0506), etomidate (Mechem Express, HY-B0100), GABA (Sigma-Aldrich, A5835) and acetylcholine chloride (Sigma-Aldrich,P6625).

### Calcium imaging

Calcium imaging was similar to previous report^36^. Individual clean 3^rd^ instar larva was briefly dissected in AHL (Adult Hemolymph-Like) solution to expose central brain, but with the anterior part of body intact. It was then transferred with AHL solution into a chamber formed by reinforcing rings on a glass slide and covered with a cover slip. The target neuron was directly localized under two-photon microscope. For calcium imaging response to light stimulation, 470 nm blue light (470 nm at peak with half-width of 10 nm) of various intensities measured at 470 nm was used for both LRIN^R13B07^s and CLPN^R82B09^s. For calcium imaging response of CLPN^R82B09^s to optogenetic inhibition of LRIN^R13B07^s using NpHR, 540 nm green light was used. All Ca^2+^ imaging experiments with light stimulation were performed with an Olympus FV-1000 two-photon microscope with ×40 water immersion lens. Infrared laser at 910 nm was used for excitation of GCAMP. The change in fluorescent intensity in neurons was captured by two-photon scanning at frequency of 2.33 frames per second and resolution of 256 × 256. For calcium imaging response of CLPN^R82B09^s to drugs, experiments were performed with an Olympus FV-1000 confocal microscope with ×10 lens. Change in fluorescent intensity was captured at frequency of 1.75 frames per second and resolution of 320 × 320.

For quantitative analysis of Ca^2+^ imaging data, ImageJ (https://imagej.nih.gov/ij/) was used to batch process images to determine fluorescence intensity of regions of interest, i.e. the cell bodies of neurons. Average fluorescence intensity (F) in 20 sequential images before stimulation or drug application was used as the basal level. Changes in fluorescence intensity (*Δ*F) were calculated and *Δ*F/F was used to indicate Ca^2+^ responses. Specifically when measuring CLPN^R82B09^s’ response to light, only responses of more than 20% increase in fluorescent intensity were considered as valid to overcome the possible effect of spontaneous oscillation.

### Immunochemistry and confocal microscopy

Third instar larval brains were dissected from larvae in PBS, fixed in PBS containing 4% paraformaldehyde for 1 h at room temperature and washed 4 × 30 min in PBS containing 0.5% Triton X-100 (PBT) before being blocked for 2 h in PBT containing 5% goat serum. The samples were then incubated with primary antibodies (rabbit anti-GABA, 1:50, Cat. A2052, Sigma-Aldrich; rabbit anti-RFP 1:100, Cat. ab62341, Abcam; mouse anti-rCD2, 1:1000, Cat. 201305, Biolegend; rabbit anti-CD4, 1:200, Cat. ab133616, Abcam; mouse anti-PDF, 1:100, PDF-C7 concentrate, DSHB; mouse anti-Fas II, 1:100, 1D4 concentrate, DSHB; mouse anti-ChAT, 1:100, ChAT4B1 concentrate, DSHB; rat anti-HA, 1:200, Cat. 11867423001, Roche) overnight at 4 °C, before being washes in PBT for 4 × 30 min. Specifically, as *QUAS-mtdTomato(3xHA)* was the designed reporter for trans-Tango signal, we used anti-HA to visualize the trans-Tango signal. The samples were then incubated with secondary antibody (Alexa 647-conjugated goat anti-rabbit, 1:100, Cat. A27040, Alexa 647-conjugated goat anti-mouse, 1:100, Cat. A21235, Alexa 647-conjugated goat anti-rat, 1:100, Cat. A21247, or Dylight 594-conjugated goat anti-rat, 1:100, Cat. SA5–10020, all from Thermo Fisher) for 2 h at room temperature and washed in PBT 3 × 10 min in darkness before being mounted and viewed. Images were acquired using an Olympus FV-1000 confocal laser scanning microscope and subsequently processed with ImageJ (www.nih.gov/ij). Specifically for processing the images about GRASP between LRIN^R13B07^s and CLPN^R82B09^s, the GRASP signal was confirmed by continuously tracing the anti-CD4 signals that marked the morphology of LRIN^R13B07^ and CLPN^R82B09^, so that they were separated from the non-specific GFP signals.

### Larval head cast analysis

The larval behavioral details in light spot assay were analyzed with a modified version of SOS^46^. In brief, single larva was sketched out from background and thinned to a line by algorithms implanted in matlab (Mathworks Inc.). The two ends of the line, head, and tail, together with midpoint of the line and centroid obtained from the larval body outline, were used to calculate headspeed, tailspeed, midspeed, and cmspeed. The bending angle of larval body, headtheta, was calculated from the angle between head-midpoint line and midpoint-tail line. The angular speed of headtheta is termed headomega.

For larval head cast size upon light-on and light-off in light spot assay, the size of head cast upon light-on was defined as previously described with modifications^46^. As larva head cast happened always after deceleration, they were considered as an assembly when we judged the light related events. First, periods in tailspeed was defined according to the fluctuation of tailspeed during larval peristalsis. Deceleration was defined if the minimum tailspeed in one period was lower than in the previous period for more than 15% and the maximum tailspeed was no higher than in the previous period. Multiple continuous deceleration periods joined into one deceleration segment. Second, the time window for picking the deceleration related head cast was defined in either of the following ways: if larval tailspeed dropped to a level below arbitrarily set threshold, the time window was the whole-subthreshold period; if the larval tailspeed after deceleration was above the threshold, the time window was from 1 tailspeed period before to 2 tailspeed periods after the end of the deceleration. Third, the largest head cast size in the time window that began within 2 tailspeed periods before or 4 tailspeed periods after larva entered light spot was picked as the size of head cast upon light-on. For head cast size upon light-off, the largest head cast size in the time window of 0.5–5 s after larva exit the light spot, or in the time window from 0.5 s after light exit to the re-entry of light if the re-entry was within 5 s after light exit, was picked as the size of head cast upon light off. The extracted values of head cast sizes were confirmed by reviewing the videos. Head cast data that didnot match with the videos due to improper image processing were discarded.

Size of larval head cast in response to optogenetic stimulation of CLPN^R82B09^s was extracted in a similar way as in response to light-on in light spot assay, except that a 5 s time window for measuring head cast size was set as from the beginning to 4 s after the ending of the 1 s red light stimulation

The codes are available at http://www.github.com/zfgong/zpp.

### Statistics

Statistics was performed with prism6.0 (Graphpad Inc.). Fisher’s exact test was used for comparing the rate of event occurrence between two groups. For comparing of event occurrence rate among three groups, we made use of the rcompanion package of R (version 3.5.0) and did *χ*^2^-test. The *p*-values between subgroup comparisons were adjusted using FDR (false discovery rate) controlled by the Benjamini-Hochberg method. For all the rest, *t*-test or one-way ANOVA with Tukey’s post hoc test were used. Error bars in scatter plot and shaded areas flanking curves represented SEM. The details of the statistics for relevant figure panels are in Supplementary Summary of Statistics.

## Supplementary information

Supplementary Information

Supplementary Movie 1

Supplementary Data 1

## Data Availability

The authors declare that the data supporting the findings of this study are available within the paper and its supplementary information files. The source data underlying all plotted Figures and Supplementary Figures are provided as a Source Data file.
