# Author Correction: A disinhibitory mechanism biases *Drosophila* innate light preference

**DOI:** 10.1038/s41467-019-08511-8

**Published:** 2019-01-29

**Authors:** Weiqiao Zhao, Peipei Zhou, Caixia Gong, Zhenhuan Ouyang, Jie Wang, Nenggan Zheng, Zhefeng Gong

**Affiliations:** 1 Department of Neurology of the Second Affiliated Hospital, Zhejiang University School of Medicine, Hangzhou, Zhejiang 310058 China; 2 Department of Neurobiology, Key Laboratory of Medical Neurobiology of the Ministry of Health of China, Key Laboratory of Neurobiology, Zhejiang University School of Medicine, Hangzhou, Zhejiang 310058 China; 3 Qiushi Academy for Advanced Studies, Zhejiang University, Hangzhou, Zhejiang 310007 China

Correction to: *Nature Communications* 10.1038/s41467-018-07929-w; published online 10 January 2019.

The original version of this Article contained an error in the author affiliations.

Affiliation 2 incorrectly read ‘Department of Neurology of the Second Affiliated Hospital, Department of Neurobiology, Key Laboratory of Medical Neurobiology of the Ministry of Health of China, Key Laboratory of Neurobiology, Zhejiang University School of Medicine, Hangzhou, Zhejiang 310007, China’ and affiliation 3 incorrectly read ‘Qiushi Academy for Advanced Studies, Zhejiang University, Hangzhou, Zhejiang 310058, China.’

This has now been corrected in both the PDF and HTML versions of the Article.

