# Supplementary material for: A disinhibitory mechanism biases Drosophila innate light preference
Source: Nat Commun. 2019 Jan 10;10:124. doi: 10.1038/s41467-018-07929-w (PMC6328558; doi:10.1038/s41467-018-07929-w)
Supplement: Supplementary file 1 — Supplementary Information [file 41467_2018_7929_MOESM1_ESM.pdf]

A disinhibitory mechanism biases *Drosophila* innate light preference

Zhao et al.

Supplementary Information

Supplementary Figures and Legends

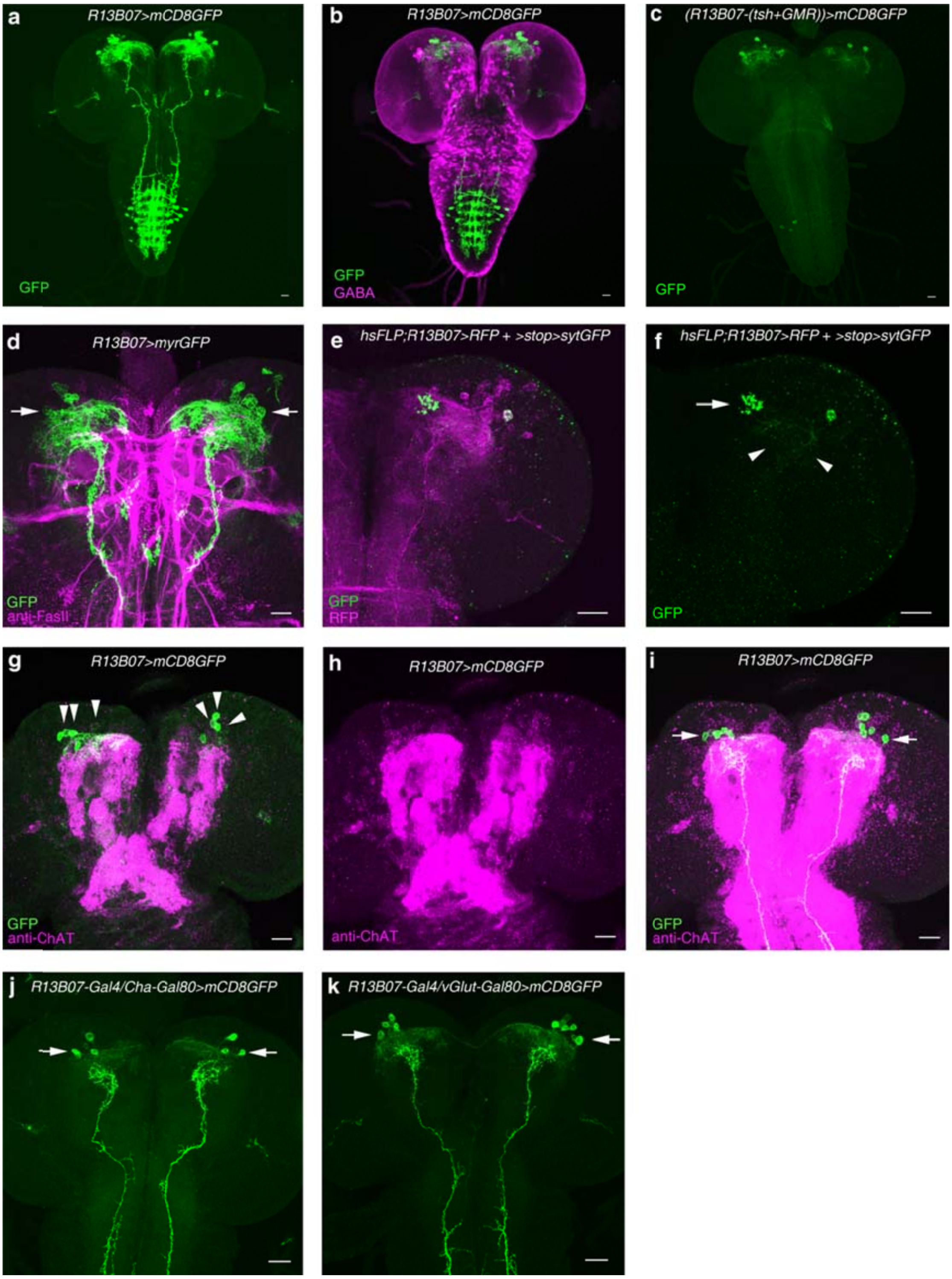

Supplementary Fig. 1 | *R13B07-Gal4* labeled by Neurons are GABA negative except for LRIN^{R13B07}s. a. Expression of *R13B07-Gal4* in larval CNS. **b**. Stacked image of GABA staining against *R13B07-Gal4* in larval VNC. **c**. *R13B07-Gal4* signal in VNC is repressed by *tsh-Gal80*. *(R13B07-(tsh+GMR))>mCD8GFP* is the short for *tsh-Gal80/UAS-mCD8GFP; R13B07-Gal4/GMR-Gal80*. **d**. Counter staining of anti-FasII against *R13B07-Gal4* labeled by

myr-GFP. Anti-FasII signal is in magenta. GFP is in green. Arrows point to cell bodies of LRIN^{R13B07}s. **e-f**. Polarity of LRIN^{R13B07}. **e** is the overlay of single LRIN^{R13B07} labeled by sytGFP and *R13B07-Gal4* labeled by RFP. **f** is the channel for single LRIN ^{R13B07}. Arrow indicates the axonal termini of LRIN^{R13B07} that is marked by sytGFP. Arrow heads indicate the dendrites of LRIN^{R13B07}. *hsFLP;R13B07>RFP+>stop>sytGFP* is the short for *hsFLP/+; UAS-RFP/+; R13B07-Gal4/UAS-FRT-stop-FRT-sytGFP*. **g**. Some of the *R13B07-Gal4* labeled neurons are cholinergic. Only parts of confocal layers are stacked to expose the co-localization signals. Arrow heads point to co-localization of *R13B07-Gal4* labeled neurons with anti-ChAT. Anti-ChAT is in magenta. GFP is in green. **h**. Magenta channel of **g**. **i**. LRIN^{R13B07}s are not cholinergic. All confocal layers are stacked. Cell bodies of LRIN^{R13B07}s are indicated by arrows. Anti-ChAT is in magenta. GFP is in green. **j**. *Cha-Gal80* represses *R13B07-Gal4* expression in larval brain. Arrows point to cell bodies of LRIN^{R13B07}s. **k**. *vGlut-Gal80* does not obviously repress *R13B07-Gal4* in larval brain. Arrows point to cell bodies of LRIN^{R13B07}s. Scale bars in all panels, 20μm.

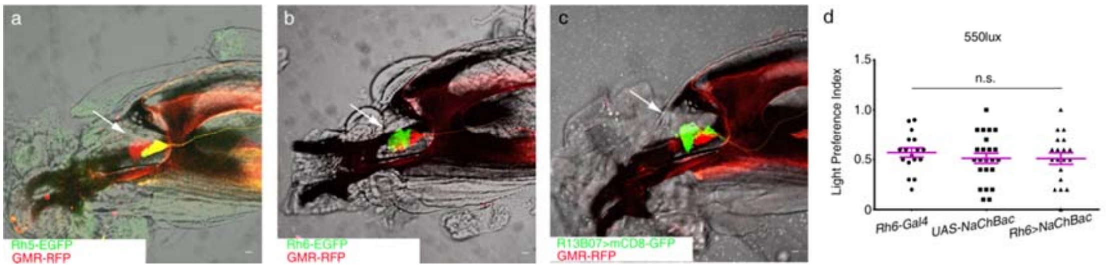

Supplementary Fig. 2 | *R13B07-Gal4* labels Rh6 positive photoreceptors. a-c. Compared with *GMR-RFP* that labels all the photoreceptors including Rh5-positive **(a)** and Rh6-positive **(b)** photoreceptors, *R13B07-Gal4* labels the Rh6-positive photoreceptors **(c)**. Arrows point to Bolwig's Organs. Scale bars in **a-c**, 20μm**. d.** Hyperactivation of *Rh6-Gal4* positive neurons does not significantly affect larval light avoidance at 550lux (23.3μW/mm^2). n.s., not significant, one way ANOVA. Error bars, SEMs.

Source data of **(d)** are provided as a source data file.

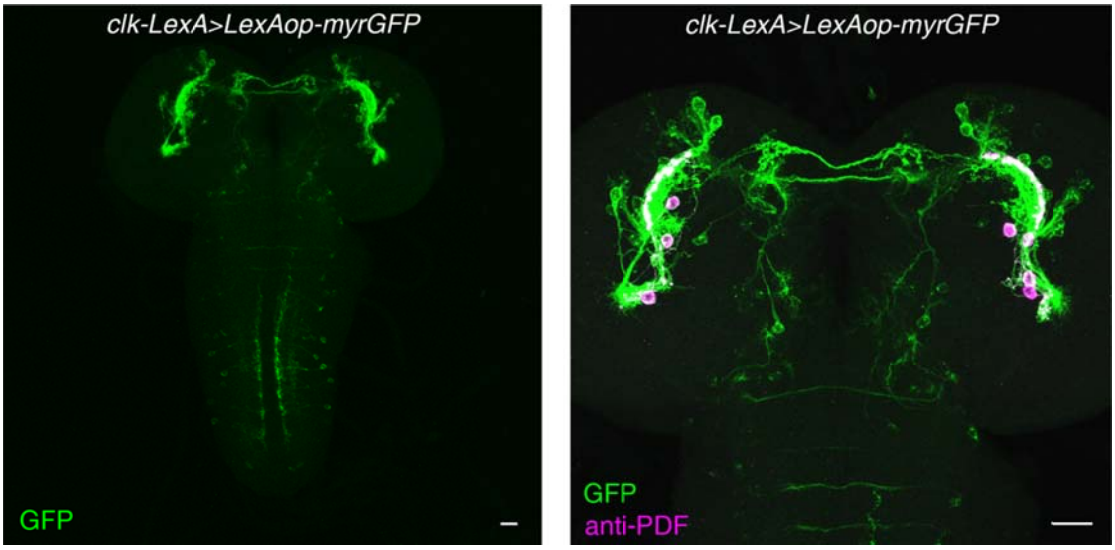

Supplementary Fig. 3 | Expression pattern of *clk-LexA* in larval CNS. Left, *clk-LexA* driven myrGFP expression in larval CNS. Right, counter staining *clk-LexA* labeled by myrGFP with anti-PDF in larval brain hemispheres. Anti-PDF is in magenta. GFP is in green. Scale bars, 20 um.

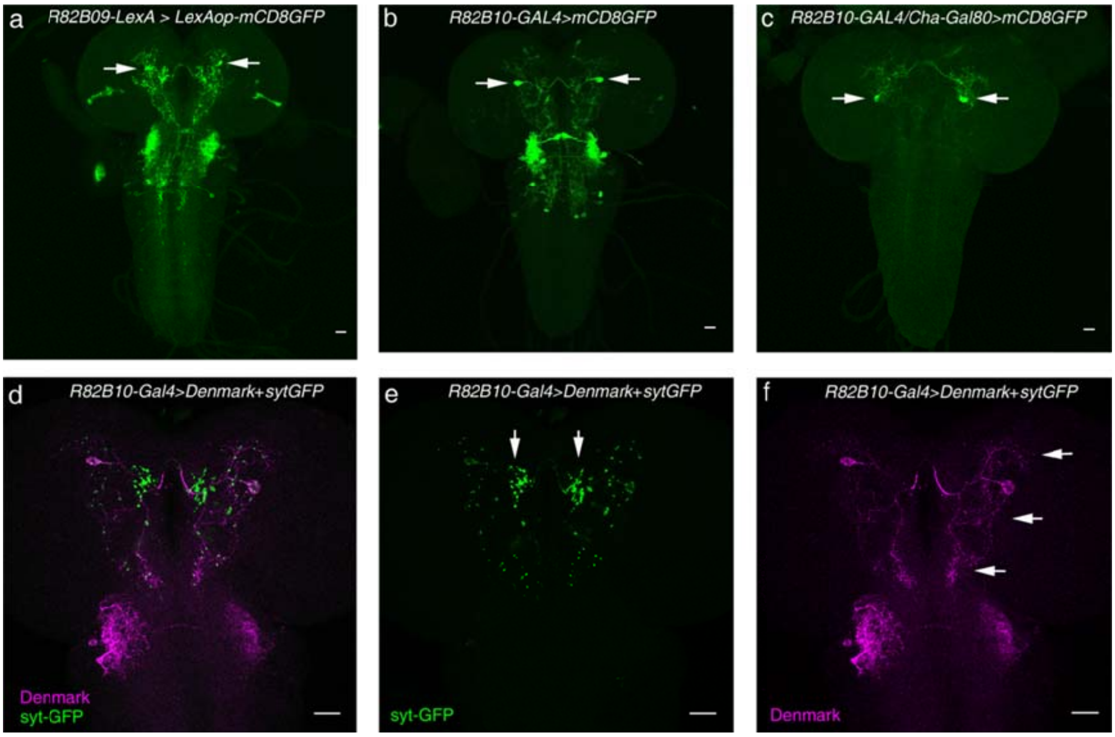

Supplementary Fig. 4 | *R82B09-LexA* and *R82B10-Gal4* label CLPN^{R82B09}s in larval CNS. a. *R82B09-LexA* labeled by mCD8GFP in larval CNS. **b-c.** *Cha-Gal80* represses *R82B10-Gal4* activity in most neurons except for CLPN^{R82B09}s. **b.** Expression pattern of *R82B10-Gal4* in larval CNS. **c.** *Cha-Gal80* removed all *R82B10-Gal4* driven GFP signal except in CLPN^{R82B09}s. Arrows point to cell bodies of CLPN^{R82B09}s in **a**-**c**. **d-f.** Pre- and post-synaptic sites of CLPN^{R82B09}s

indicated by syt-GFP and Denmark respectively. **d** is the composite of **e** and **f**, which are the channels for syt-GFP and Denmark respectively. Arrows point to presynaptic sites in **e** and postsynaptic sites in **f**. Scale bars in all panels, 20μm.

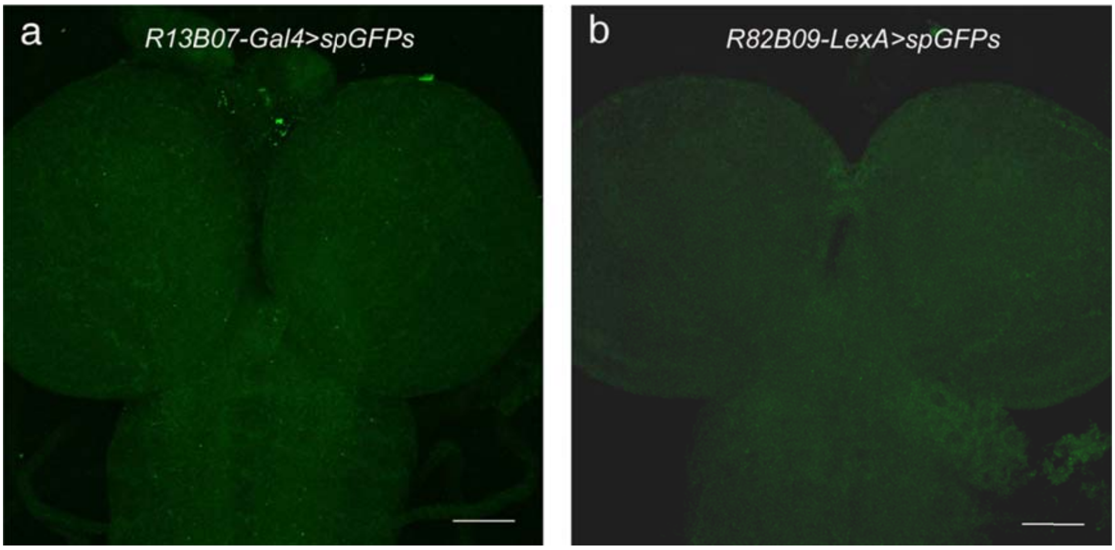

Supplementary Fig. 5 | Controls of GRASP. Driving expression of spGFPs (*UAS-spGFP*$_{1\text{-}10}$*;LexAop-GFP*$_{11}$) with *R13B07-Gal4* (**a**) or *R82B09-LexA* (**b**) alone does not produce GRASP signal at the putative overlapping site of LRIN^{R13B07}s and CLPN^{R82B09}s. Scale bars in all panels, 20μm.

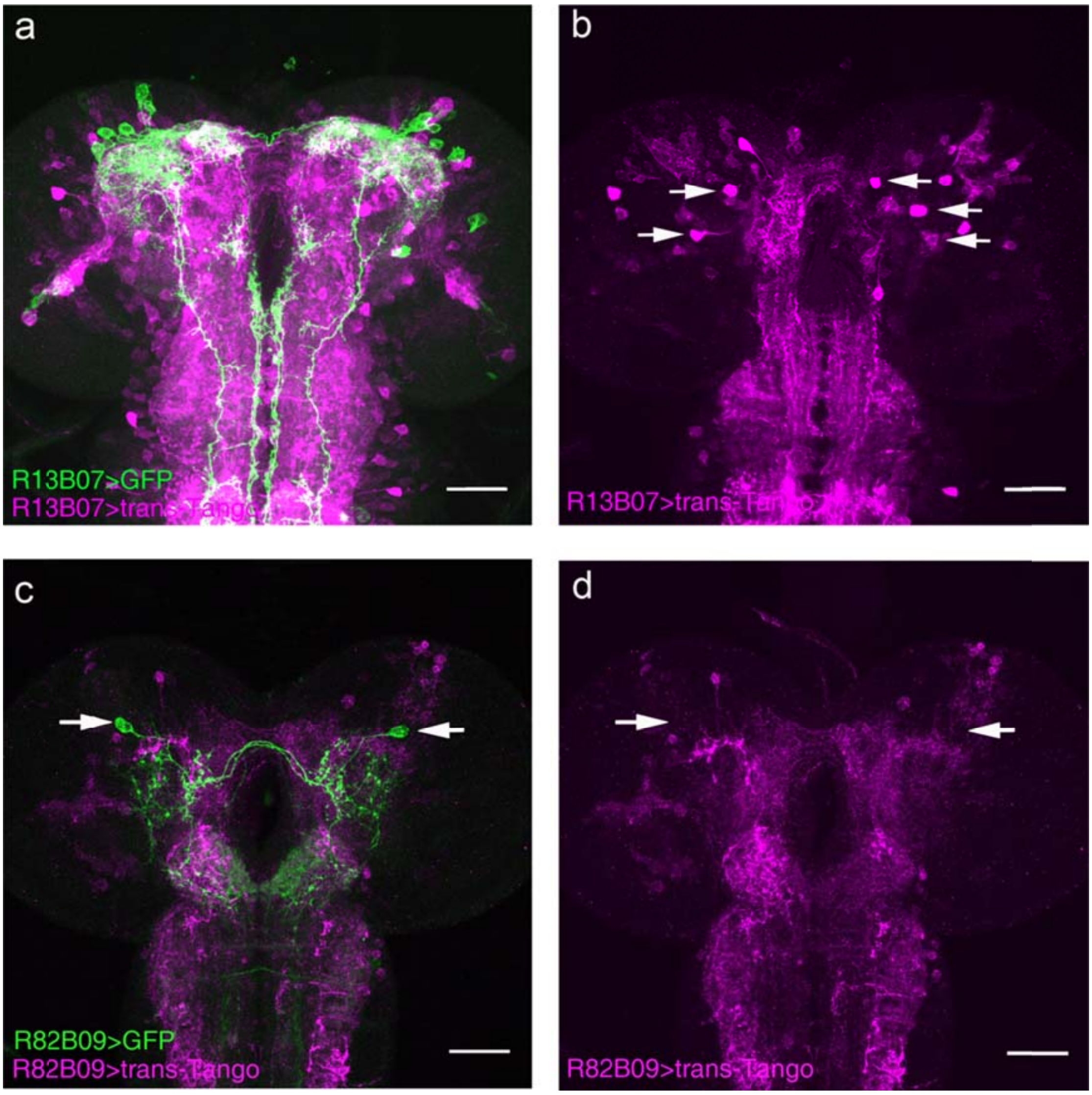

Supplementary Fig. 6 | Controls trans-Tango. a-b. Driving trans-Tango expression with *R13B07-Gal4* produces signals that resemble CLPN^{R82B09}s. **a** is stack of all confocal layers. **b** is the stack of selected slices that putatively include the layers of CLPN^{R82B09}s. Arrows in **b** indicate cell bodies that could be CLPN^{R82B09}s. **c-d**. Driving trans-Tango expression with *R82B09-Gal4* alone does not reveal any downstream signal that overlaps with GFP-labeled CLPN^{R82B09}s. Only confocal layers at levels of CLPN^{R82B09}s' cell bodies are stacked. trans-Tango signal is in magenta. GFP signal is in green. Arrows indicate cell bodies of CLPN^{R82B09}s. The genotypes of *UAS-myrGFP,QUAS-mtdTomato(3xHA)/+;trans-Tango/+;R13B07-Gal4/+* and *UAS-myrGFP,QUAS-mtdTomato(3xHA)/+;trans-Tango/+;R82B09-Gal4/+* were used in **a-b** and **c-d** respectively. trans-Tango signals are reported by 3xHA. Scale bars in all panels, 20μm.

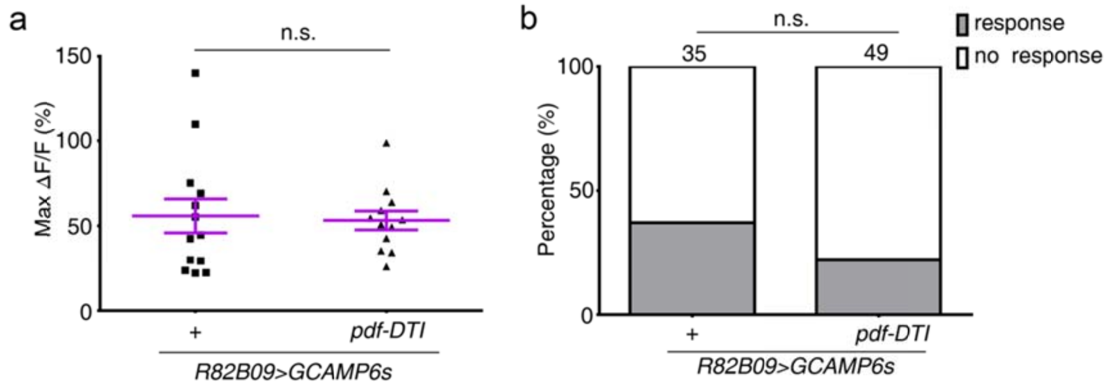

Supplementary Fig. 7 | Ablating *pdf* neurons does not significantly affect CLPN^{R82B09}s' response to light. a. Ablating *pdf* neurons does not affect the amplitude of CLPN^{R82B09}s' response to light in calcium imaging. **b**. Ablating *pdf* neurons does not affect the probability for CLPN^{R82B09}s to respond to light in calcium imaging. 470nm light at intensity of 1.058μW/mm^2 was used for 1 second. Numbers above bars indicate sample sizes. Genotypes of lines used are *pdf-DTI/R82B09-LexA; LexAop-GCAMP6s/+* and *R82B09-LexA/+;LexAop-GCAMP6s/+*. n.s. not significant, *t*-test in **a**, *fisher's* exact test in **b**. Error bars in **a**, SEMs.

Source data of (**a-b**) are provided as a source data file.

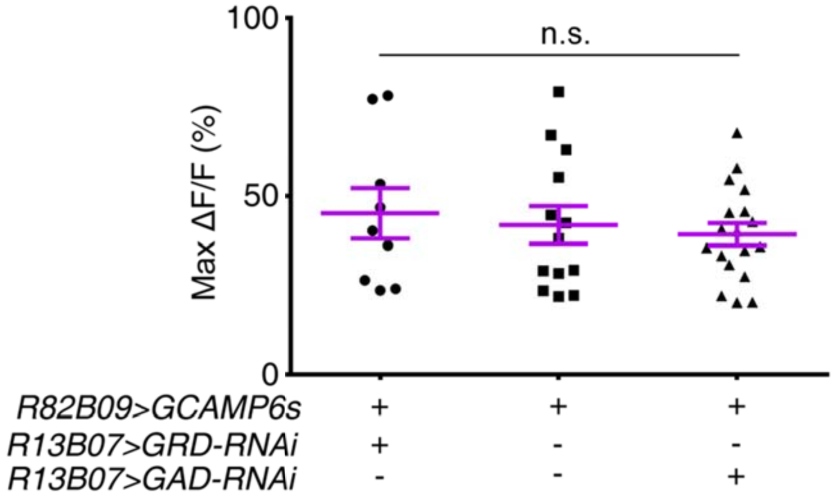

Supplementary Fig. 8 | Knocking down GRD and GAD expression in LRIN^{R13B07}s does not affect the amplitude of CLPN^{R82B09}s' response to light. 470nm light at intensity of 1.058μW/mm^2 was used for 1 second. Genotypes of larvae used are *R82B09-LexA/UAS-GRD-RNAi;LexAop-GCAMP6s/R13B07-Gal4*, *R82B09-LexA/+;LexAop-GCAMP6s/+* and

R82B09-LexA/UAS-GAD-RNAi;LexAop-GCAMP6s/R13B07-Gal4 respectively. n.s. not significant, one way ANOVA. Error bars, SEMs.

Source data are provided as a source data file.

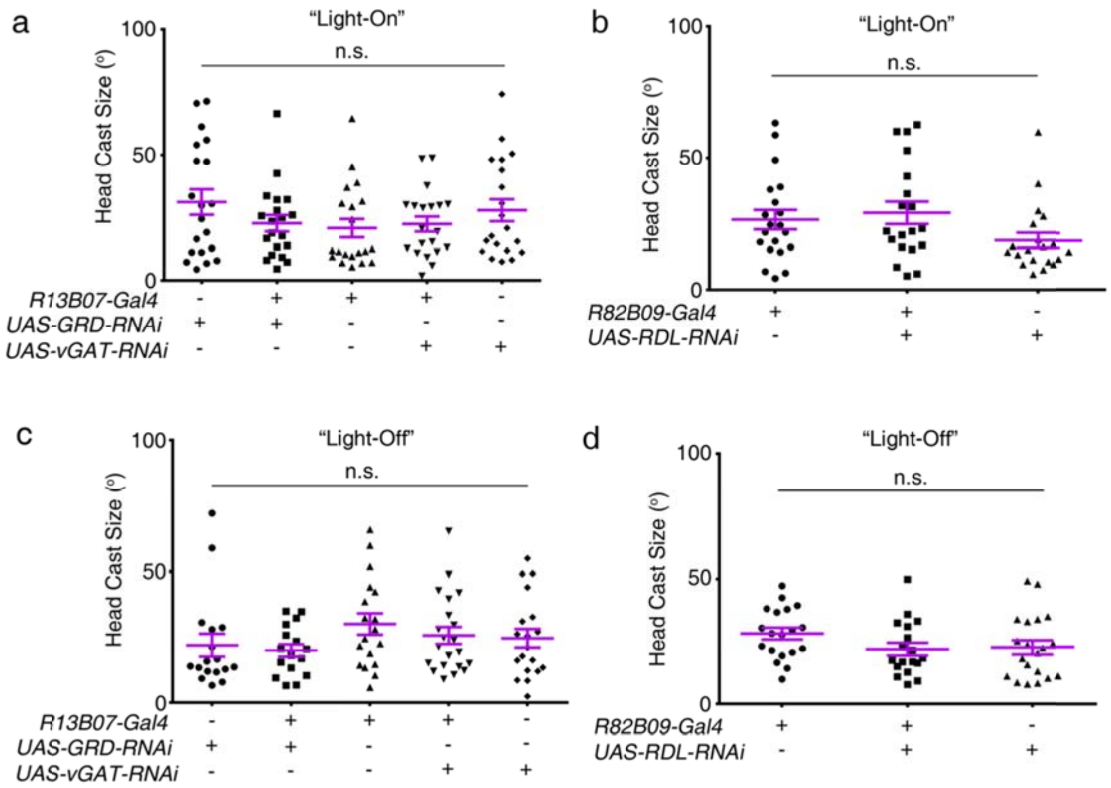

Supplementary Fig. 9 | Disturbing the LRIN^{R13B07}s-CLPN^{R82B09}s disinhibitory circuit does not significantly affect larval head cast in darkness. a-b. Size of larval head cast at "light-on" in darkness is not significantly affected by down regulation of GRD or vGAT in LRIN^{R13B07}s (**a**), or by down regulation of RDL in CLPN^{R82B09}s (**b**). **c-d**. Size of larval head cast at "light-off" in darkness is not significantly affected by down regulation of GRD or vGAT in LRIN^{R13B07}s (**c**), or by down regulation of RDL in CLPN^{R82B09}s (**d**). The light spot assay was performed with the light constantly turned off. n.s. not significant, one way ANOVA. Error bars, SEMs.

Source data of (**a-d**) are provided as a source data file.

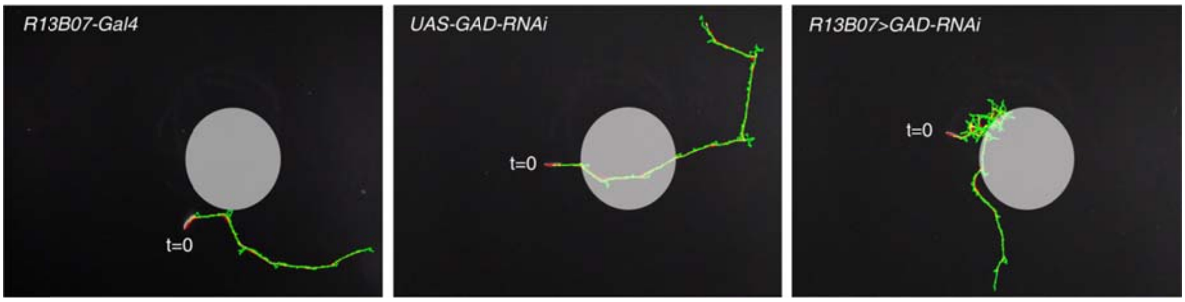

Supplementary Fig. 10 | Down-regulation of GAD in LRIN^{R13B07}s improves the chance for larva to return to light spot after the initial escape. Three representative trajectories are shown. Red, yellow and green represent tracks of tail, midpoint and head of larva. The starting points are indicated by "t = 0".
