# Supplementary material for: A disinhibitory mechanism biases Drosophila innate light preference
Source: Nat Commun. 2019 Jan 10;10:124. doi: 10.1038/s41467-018-07929-w (PMC6328558; doi:10.1038/s41467-018-07929-w)
Supplement: Supplementary file 3 — Supplementary Data 1 [file 41467_2018_7929_MOESM3_ESM.docx]

**Supplementary Summary of Statistics**

**Figure 1**

**Figure 1a**

One-way ANOVA followed by post hoc Tukey’s multiple comparison test.

F(4,88)=38.57, P<0.0001.

| Genotype | *w1118* | *R13B07-Gal4* | *UAS-NaChBac* | *R13B07> NaChBac* | *R13B07-GAL4/tsh-Gal80>NaChBac* |
| --- | --- | --- | --- | --- | --- |
| N | 12 | 35 | 14 | 18 | 14 |
| Mean | 0.7000 | 0.4286 | 0.7071 | 0.03976 | 0.06429 |
| SEM | 0.05505 | 0.02970 | 0.04857 | 0.03591 | 0.08491 |

| comparison | *w1118*  vs  *R13B07>NaChBac* | *R13B07-Gal4*  *vs*  *R13B07>NaChBac* | *UAS-NaChBac/+*  *vs*  *R13B07>NaChBac* | *R13B07>NaChBac*  *vs*  *R13B07-GAL4/tsh-Gal80>NaChBac* |
| --- | --- | --- | --- | --- |
| p-value | <0.0001 | <0.0001 | <0.0001 | 0.9970 |

**Figure 1e**

unpaired t-test, two-tailed.

| Group | light | No light |
| --- | --- | --- |
| N | 7 | 7 |
| Mean | -24.93 | -8.877 |
| SEM | 2.118 | 2.566 |
| p-value | 0.0004 | - |
| t_[12]_ | 4.824 | - |

**Figure 1g**

One-way ANOVA.

F(2,25)=48.33, P<0.0001.

| Group | 0.014μW/mm^2^ | 1.058μW/mm^2^ | 105.8μW/mm^2^ |
| --- | --- | --- | --- |
| N | 7 | 10 | 11 |
| Mean | -11.76 | -27.86 | -41.51 |
| SEM | 1.618 | 2.370 | 1.836 |

**Figure 1h**

One-way ANOVA followed by post hoc Tukey’s multiple comparison test.

F(2,27)=11.74, P<0.001.

| Group | *pdf-DTI;R13B07>GCAMP6.0* | *R13B07>GCAMP6.0* | *R13B07>GCAMP6.0; clk>TNTG* |
| --- | --- | --- | --- |
| N | 13 | 9 | 8 |
| Mean | -27.19 | -32.77 | -20.78 |
| SEM | 1.306 | 1. 627 | 2.084 |

| comparison | *R13B07>GCAMP6.0*  vs  *pdf-DTI;R13B07>GCAMP6.0* | *R13B07>GCAMP6.0*  vs  *R13B07>GCAMP6.0; clk>TNTG* | *pdf-DTI;R13B07>GCAMP6.0*  vs  *R13B07>GCAMP6.0; clk>TNTG* |
| --- | --- | --- | --- |
| p-value | 0.0454 | 0.0001 | 0.0243 |

**Figure 1i**

One-way ANOVA followed by post hoc Tukey’s multiple comparison test.

F(2,39)=15.88, P<0.0001.

| Group | *R13B07>GCAMP6.0* | *R13B07>GCAMP6.0*+*RDL-RNAi* | *R13B07>GCAMP6.0*+*GRD-RNAi* |
| --- | --- | --- | --- |
| N | 13 | 14 | 15 |
| Mean | -29.06 | -35.51 | -16.44 |
| SEM | 1.260 | 3.539 | 1.226 |

| comparison | *R13B07>GCAMP6.0*  vs  *R13B07>GCAMP6.0*+*RDL-RNAi* | *R13B07>GCAMP6.0*  vs  *R13B07>GCAMP6.0*+*GRD-RNAi* |
| --- | --- | --- |
| p-value | 0.1354 | 0.0010 |

**Figure 1l**

One-way ANOVA followed by post hoc Tukey’s multiple comparison test.

F(4,86)=12.77, P<0.0001.

| Group | *UAS-GAD-RNAi* | *R13B07> GAD-RNAi* | *R13B07-GAL4* | *R13B07>vGAT-RNAi* | *UAS-vGAT-RNAi* |
| --- | --- | --- | --- | --- | --- |
| N | 16 | 17 | 23 | 19 | 16 |
| Mean | 0.1894 | 0.5479 | 0.2264 | 0.5542 | 0.2313 |
| SEM | 0.05207 | 0.04482 | 0.05473 | 0.05167 | 0.04539 |

| comparison | *UAS-GAD-RNAi*  *vs*  *R13B07> GAD-RNAi* | *R13B07> GAD-RNAi*  *vs*  *R13B07-GAL4* | *R13B07-GAL4*  *vs*  *R13B07>vGAT-RNAi* | *R13B07>vGAT-RNAi*  *vs*  *UAS-vGAT-RNAi* |
| --- | --- | --- | --- | --- |
| p-value | <0.0001 | 0.0001 | <0.0001 | 0.0004 |

**Figure 2**

**Figure 2b**

unpaired t-test, two-tailed.

| Group | *UAS-NpHR;R82B09>GCAMP6.0* | *R13B07>NpHR;R82B09>GCAMP6.0* |
| --- | --- | --- |
| N | 8 | 8 |
| Mean | 6.981 | 26.80 |
| SEM | 1.620 | 2.954 |
| p-value | <0.0001 | - |
| t_[14]_ | 5.883 | - |

**Figure 2k**

unpaired t-test, two-tailed.

| Group | Ach | Ach+GABA |
| --- | --- | --- |
| N | 5 | 7 |
| Mean | 95.21 | 14.57 |
| SEM | 25.21 | 2.633 |
| p-value | 0.0034 | - |
| t_[10]_ | 3.820 | - |

**Figure 2m**

unpaired t-test, two-tailed.

| Group | saline | PTX |
| --- | --- | --- |
| N | 7 | 7 |
| Mean | 19.17 | 139.5 |
| SEM | 7.267 | 30.39 |
| p-value | 0.0023 | - |
| t_[12]_ | 3.850 | - |

**Figure 2p**

unpaired t-test, two-tailed.

| Group | saline | etomidate |
| --- | --- | --- |
| N | 7 | 4 |
| Mean | -14.74 | -36.29 |
| SEM | 4.916 | 5.355 |
| p-value | 0.0208 | - |
| t_[9]_ | 2.798 | - |

**Figure 3**

**Figure 3a**

One-way ANOVA followed by post hoc Tukey’s multiple comparison test.

F(6,107)=20.30, P<0.0001.

| Group | *R82B09-GAL4* | *R82B09> TNTG* | *UAS-TNTG* | *R82B10> TNTG* | *R82B10-GAL4* | *R82B10-GAL4/Cha-Gal80>UAS-TNTG* | *UAS-TNTG/+;Cha-Gal80/+* |
| --- | --- | --- | --- | --- | --- | --- | --- |
| N | 12 | 22 | 14 | 14 | 14 | 20 | 18 |
| Mean | 0.5500 | 0.1818 | 0.4929 | -0.1500 | 0.4786 | 0.1150 | 0.6611 |
| SEM | 0.06093 | 0.06535 | 0.05494 | 0.08817 | 0.04937 | 0.05679 | 0.05122 |

| comparison | *R82B09-GAL4*  *vs*  *R82B09-GAL4>UAS-TNTG* | *R82B09> TNTG*  *vs*  *UAS-TNTG* | *R82B10> TNTG*  *vs*  *UAS-TNTG* | *R82B10> TNTG*  *vs*  *R82B10-GAL4* | *R82B10-GAL4*  *vs*  *R82B10-GAL4/ Cha-Gal80>UAS-TNTG* | *R82B10-GAL4>UAS-TNTG;Cha-Gal80*  *vs*  *UAS-TNTG/+;Cha-Gal80/+* |
| --- | --- | --- | --- | --- | --- | --- |
| p-value | 0.0019 | 0.0090 | <0.0001 | <0.0001 | 0.0015 | <0.0001 |

**Figure 3b**

One-way ANOVA followed by post hoc Tukey’s multiple comparison test.

F(4,83)=10.29, P<0.0001.

| Group | *R82B09-Gal4* | *R82B09>RDL-RNAi* | *RDL-RNAi* | *R82B10>RDL-RNAi* | *R82B10-Gal4* |
| --- | --- | --- | --- | --- | --- |
| N | 16 | 23 | 16 | 15 | 18 |
| Mean | 0.1313 | 0.3426 | 0.04438 | 0.3687 | 0.1722 |
| SEM | 0.05141 | 0.04600 | 0.03876 | 0.04343 | 0.02778 |

| comparison | *R82B09-Gal4*  *vs R82B09>RDL-RNAi* | *R82B09>RDL-RNAi*  *vs*  *RDL-RNAi* | *RDL-RNAi*  *vs*  *R82B10>RDL-RNAi* | *R82B10>RDL-RNAi*  *vs*  *R82B10-Gal4* |
| --- | --- | --- | --- | --- |
| p-value | 0.0048 | <0.0001 | <0.0001 | 0.0206 |

**Figure 3d**

One-way ANOVA followed by post hoc Tukey’s multiple comparison test.

F(3,45)=28.20, P<0.0001.

| Group | *w^1118^* | *R82B10-GAL4* | *UAS-Chrimson/+* | *R82B10>Chrimson* |
| --- | --- | --- | --- | --- |
| N | 10 | 13 | 16 | 10 |
| Mean | 10.85 | 25.78 | 21.19 | 67.57 |
| SEM | 0.9398 | 3.141 | 4.136 | 7.082 |

| comparison | *w^1118^*  *vs*  *R82B10>Chrimson* | *R82B10-GAL4*  *vs*  *R82B10>Chrimson* | *UAS-CsChrimson/+*  *vs*  *R82B10>Chrimson* |
| --- | --- | --- | --- |
| p-value | <0.0001 | <0.0001 | <0.0001 |

**Figure 3f**

One-way ANOVA followed by post hoc Tukey’s multiple comparison test.

F(2,30)=17.71, P<0.0001.

| Group | *R82B09>GCAMP6.0* + no light | *R82B09>GCAMP6.0* + light | *R82B09>GCAMP6.0+RDL-RNAi* + light |
| --- | --- | --- | --- |
| N | 5 | 14 | 14 |
| Mean | 4.120 | 38.83 | 56.98 |
| SEM | 1.466 | 3.811 | 5.816 |

| Group | *R82B09>GCAMP6.0* + no light  vs  *R82B09>GCAMP6.0* + light | *R82B09>GCAMP6.0* + light  vs  *R82B09>GCAMP6.0+RDL-RNAi* + light |
| --- | --- | --- |
| p-value | 0.0015 | 0.0236 |

**Figure 3g**

Fisher’s exact test.

P= 0.0024

| Group | *R82B09>GCAMP6.0s* | *R82B09>GCAMP6.0s*+*RDL-RNAi* |
| --- | --- | --- |
| Response | 15 | 14 |
| No response | 58 | 12 |
| p-value | 0.0024 | - |

**Figure 3h**

unpaired t-test, two-tailed.

| Group | *R82B09>GCAMP6m* | *clk>TNTG;R82B09>GCAMP6m* |
| --- | --- | --- |
| N | 11 | 4 |
| Mean | 39.64 | 37.30 |
| SEM | 5.342 | 4.362 |
| p-value | 0.8073 | - |
| t_[13]_ | 0.2489 | - |

**Figure 3i**

Fisher’s exact test.

P= 0.023

| Group | *R82B09>GCAMP6m* | *clk>TNTG;R82B09>GCAMP6m* |
| --- | --- | --- |
| Response | 11 | 4 |
| No response | 36 | 55 |
| p-value | 0.023 | - |

**Figure 4**

**Figure 4a**

Chi-square test with post hoc multiple comparisons controlled by Benjamini-Hochberg method.

χ^2^ = 21.254, df = 2, P=2.425e-05.

| Group | *R13B07-Gal4>GRD-RNDi;R82B09>GCAMP6.0s* | *R82B09>GCAMP6.0s* | *R13B07-Gal4>GAD-RNDi;R82B09>GCAMP6.0s* |
| --- | --- | --- | --- |
| Response | 9 | 13 | 18 |
| No response | 59 | 29 | 13 |

| comparison | *R13B07-Gal4>GRD-RNDi;R82B09>GCAMP6.0s*  *vs*  *R82B09>GCAMP6.0s* | *R13B07-Gal4>GAD-RNDi;R82B09>GCAMP6.0s*  *vs*  *R82B09>GCAMP6.0s* |
| --- | --- | --- |
| p-value | 0.0443 | 0.0443 |

**Figure 4b**

One-way ANOVA followed by post hoc Tukey’s multiple comparison test.

F(2,120)=5.249, P=0.0065.

| Group | *R13B07-Gal4* | *R13B07>GRD-RNAi* | *UAS-GRD-RNAi* |
| --- | --- | --- | --- |
| N | 34 | 45 | 44 |
| Mean | 44.07 | 31.99 | 46.06 |
| SEM | 3.853 | 2.689 | 3.763 |

| comparison | *R13B07-Gal4*  *vs*  *R13B07>GRD-RNAi* | *R13B07>GRD-RNAi*  *vs*  *UAS-GRD-RNAi* |
| --- | --- | --- |
| p-value | 0.0442 | 0.0085 |

**Figure 4c**

One-way ANOVA followed by post hoc Tukey’s multiple comparison test.

F(4,168)=11.96, P<0.0001.

| Group | *UAS-GAD-RNAi* | *R13B07>GAD-RNAi* | *R13B07-Gal4* | *R13B07>vGAT-RNAi* | *UAS-vGAT-RNAi* |
| --- | --- | --- | --- | --- | --- |
| N | 20 | 35 | 42 | 39 | 37 |
| Mean | 25.74 | 47.26 | 29.78 | 53.88 | 33.32 |
| SEM | 4.023 | 3.422 | 3.078 | 3.761 | 2.792 |

| comparison | *UAS-GAD-RNAi*  *vs*  *R13B07>GAD-RNAi* | *R13B07>GAD-RNAi*  *vs*  *R13B07-Gal4* | *R13B07-Gal4*  *vs R13B07>vGAT-RNAi* | *R13B07>vGAT-RNAi*  *vs*  *UAS-vGAT-RNAi* |
| --- | --- | --- | --- | --- |
| p-value | 0.0017 | 0.0018 | <0.0001 | 0.0001 |

**Figure 4d**

One-way ANOVA followed by post hoc Tukey’s multiple comparison test.

F(4,141)=6.797, P<0.0001.

| Group | *R82B09-Gal4* | *R82B09>RDL-RNAi* | *UAS-RDL-RNAi* | *R82B10>RDL-RNAi* | *R82B10-Gal4* |
| --- | --- | --- | --- | --- | --- |
| N | 28 | 43 | 20 | 31 | 24 |
| Mean | 27.08 | 40.28 | 25.00 | 44.99 | 25.40 |
| SEM | 2.917 | 3.086 | 3.105 | 4.888 | 2.418 |

| comparison | *R82B09-Gal4*  *vs*  *R82B09 >RDL-RNAi* | *R82B09>RDL-RNAi*  *vs*  *UAS-RDL-RNAi* | *UAS-RDL-RNAi*  *vs*  *R82B10>RDL-RNAi* | *R82B10>RDL-RNAi*  *vs*  *R82B10-Gal4* |
| --- | --- | --- | --- | --- |
| p-value | 0.0440 | 0.0327 | 0.0039 | 0.0026 |

**Figure 5**

**Figure 5a**

One-way ANOVA followed by post hoc Tukey’s multiple comparison test.

F(2,106)=3.157, P=0.0512.

| Group | *R13B07-Gal4* | *R13B07>GRD-RNAi* | *UAS-GRD-RNAi* |
| --- | --- | --- | --- |
| N | 29 | 44 | 36 |
| Mean | 32.44 | 30.52 | 44.29 |
| SEM | 5.170 | 3.230 | 4.909 |

| comparison | *R13B07-Gal4*  *vs*  *R13B07>GRD-RNAi* | *R13B07>GRD-RNAi*  *vs*  *UAS-GRD-RNAi* |
| --- | --- | --- |
| p-value | 0.9492 | 0.0529 |

**Figure 5b**

One-way ANOVA followed by post hoc Tukey’s multiple comparison test.

F(4,141)=10.61, P<0.0001.

| Group | *UAS-GAD-RNAi* | *R13B07>GAD-RNAi* | *R13B07-Gal4* | *R13B07>vGAT-RNAi* | *UAS-vGAT-RNAi* |
| --- | --- | --- | --- | --- | --- |
| N | 16 | 28 | 33 | 32 | 37 |
| Mean | 24.82 | 45.02 | 23.25 | 50.69 | 23.36 |
| SEM | 4.136 | 4.529 | 2.400 | 5.911 | 2.946 |
| p-value | 0.9811 | 0.0156 | - | <0.0001 | >0.9999 |

| comparison | *UAS-GAD-RNAi*  *vs*  *R13B07>GAD-RNAi* | *R13B07>GAD-RNAi*  *vs*  *R13B07-Gal4* | *R13B07-Gal4*  *vs R13B07>vGAT-RNAi* | *R13B07> vGAT-RNAi*  *vs*  *UAS-vGAT-RNAi* |
| --- | --- | --- | --- | --- |
| p-value | 0.0393 | 0.0024 | <0.0001 | <0.0001 |

**Figure 5c**

One-way ANOVA followed by post hoc Tukey’s multiple comparison test.

F(4,124)=5.866, P<0.0011.

| Group | *R82B09-Gal4* | *R82B09>RDL-RNAi* | *UAS-RDL-RNAi* | *R82B10>RDL-RNAi* | *R82B10-Gal4* |
| --- | --- | --- | --- | --- | --- |
| N | 26 | 34 | 19 | 29 | 21 |
| Mean | 26.05 | 40.53 | 20.46 | 40.12 | 24.43 |
| SEM | 3.469 | 3.743 | 2.949 | 4.803 | 2.456 |
| p-value | 0.0421 | 0.0046 | - | 0.0081 | 0.0472 |

| comparison | *R82B09-Gal4*  *vs*  *R82B09 >RDL-RNAi* | *R82B09>RDL-RNAi*  *vs*  *UAS-RDL-RNAi* | *UAS-RDL-RNAi*  *vs*  *R82B10>RDL-RNAi* | *R82B10>RDL-RNAi*  *vs*  *R82B10-Gal4* |
| --- | --- | --- | --- | --- |
| p-value | 0.0440 | 0.0327 | 0.0039 | 0.0026 |

**Figure 5d**

Chi-square test with post hoc multiple comparisons controlled by Benjamini-Hochberg method.

χ^2^ = 13.443, df = 2, P=0.001205.

| Group | *R13B07-Gal4* | *UAS-GAD-RNAi* | *R13B07 >GAD-RNAi* |
| --- | --- | --- | --- |
| Response | 3 | 6 | 16 |
| No response | 37 | 34 | 25 |

| comparison | *R13B07-Gal4*  *vs*  *R13B07 >GAD-RNAi* | *UAS-GAD-RNAi*  *vs*  *R13B07 >GAD-RNAi* |
| --- | --- | --- |
| p-value | 0.00609 | 0.0438 |

**Figure 5e**

Chi-square test with post hoc multiple comparisons controlled by Benjamini-Hochberg method.

χ^2^ = 10.879, df = 2, P=0.004341.

| Group | *R82B09-Gal4* | *UAS-RDL-RNAi* | *R82B09 >RDL-RNAi* |
| --- | --- | --- | --- |
| Response | 5 | 5 | 16 |
| No response | 55 | 55 | 44 |

| comparison | *R82B09-Gal4*  *vs*  *R82B09 >RDL-RNAi* | *UAS-RDL-RNAi*  *vs*  *R82B09 >RDL-RNAi* |
| --- | --- | --- |
| p-value | 0.0244 | 0.0244 |

**Supplementary Figures**

**Supplementary Figure 2**

**Supplementary Figure 2d**

One-way ANOVA.

F(2,56) = 0.5019, P=0.6081.

| Group | *Rh6-Gal4* | *UAS-NaChBac* | *Rh6>NaChBac* |
| --- | --- | --- | --- |
| N | 17 | 23 | 19 |
| Mean | 0.5706 | 0.5130 | 0.5105 |
| SEM | 0.04735 | 0.05081 | 0.05717 |

**Supplementary Figure 7**

**Supplementary Figure 7a**

unpaired t-test, two-tailed.

| Group | *R13B07>GCAMP6.0s* | *Pdf-DTI; R13B07>GCAMP6.0s* |
| --- | --- | --- |
| N | 13 | 12 |
| Mean | 55.94 | 53.31 |
| SEM | 9.99 | 5.55 |
| p-value | 0.824 | - |
| t_[23]_ | 0.225 | - |

Supplementary Figure 7b

Fisher’s exact test.

P = 0.2303

| Group | *R82B09>GCAMP6.0s* | *Pdf-DTI; R82B09>GCAMP6.0s* |
| --- | --- | --- |
| Response | 13 | 12 |
| No response | 22 | 38 |
| p-value | 0.2303 | - |

**Supplementary Figure 8**

One-way ANOVA.

F(2,37) = 0.3610, P=0.6994.

| Group | *R82B09>GCAMP6.0;*  *R13B07>GRD-RNAi* | *R82B09>GCAMP6.0* | *R82B09>GCAMP6.0;*  *R13B07>GAD-RNAi* |
| --- | --- | --- | --- |
| N | 9 | 13 | 18 |
| Mean | 45.27 | 42.02 | 39.40 |
| SEM | 7.022 | 5.254 | 3.101 |

**Supplementary Figure 9**

**Supplementary Figure 9a**

One-way ANOVA.

F(4,95)=1.232, P=0.3027.

| Group | *UAS-GRD-RNAi* | *R13B07>GRD-RNAi* | *R13B07-Gal4* | *R13B07>vGAT-RNAi* | *UAS-vGAT-RNAi* |
| --- | --- | --- | --- | --- | --- |
| N | 20 | 20 | 20 | 20 | 20 |
| Mean | 31.22 | 22.76 | 20.83 | 22.43 | 27.94 |
| SEM | 5.066 | 3.259 | 3.628 | 2.972 | 4.379 |

**Supplementary Figure 9b**

One-way ANOVA.

F(2,56)=2.231, P=0.1169.

| Group | *R82B09-Gal4* | *R82B09>RDL-RNAi* | *UAS-RDL-RNAi* |
| --- | --- | --- | --- |
| N | 20 | 20 | 20 |
| Mean | 31.22 | 22.76 | 20.83 |
| SEM | 5.066 | 3.259 | 3.628 |

**Supplementary Figure 9c**

One-way ANOVA.

F(4,86)=1.082, P=0.3705.

| Group | *UAS-GRD-RNAi* | *R13B07>GRD-RNAi* | *R13B07-Gal4* | *R13B07>vGAT-RNAi* | *UAS-vGAT-RNAi* |
| --- | --- | --- | --- | --- | --- |
| N | 17 | 16 | 18 | 21 | 19 |
| Mean | 22.00 | 19.98 | 30.08 | 25.71 | 24.68 |
| SEM | 4.366 | 2.394 | 4.077 | 3.244 | 3.526 |

**Supplementary Figure 9d**

One-way ANOVA.

F(2,54)=1.712, P=0.1901.

| Group | *R82B09-Gal4* | *R82B09>RDL-RNAi* | *UAS-RDL-RNAi* |
| --- | --- | --- | --- |
| N | 19 | 18 | 20 |
| Mean | 28.25 | 21.98 | 22.71 |
| SEM | 2.347 | 2.587 | 2.844 |
